# High-throughput multi-residue quantification of contaminants of emerging concern in wastewaters enabled using direct injection liquid chromatography-tandem mass spectrometry

**DOI:** 10.1016/j.jhazmat.2020.122933

**Published:** 2020-11-05

**Authors:** Keng Tiong Ng, Helena Rapp-Wright, Melanie Egli, Alicia Hartmann, Joshua C. Steele, Juan Eduardo Sosa-Hernández, Elda M. Melchor-Martínez, Matthew Jacobs, Blánaid White, Fiona Regan, Roberto Parra-Saldivar, Lewis Couchman, Rolf U. Halden, Leon P. Barron

**Affiliations:** a Dept. Analytical, Environmental & Forensic Sciences, King’s College London, 150 Stamford Street, London, SE1 9NH, United Kingdom; b DCU Water Institute and School of Chemical Sciences, Dublin City University, Glasnevin, Dublin 9, Ireland; c Hochschule Fresenius, Limburger Straße 2, Idstein, Hessen, Germany; d Biodesign Center for Environmental Health Engineering, The Biodesign Institute, Arizona State University, 1001 S. McAllister Avenue, Tempe, AZ 85287-8101, USA; e Tecnologico de Monterrey, Escuela de Ingenieria y Ciencias, Campus Monterrey, Ave. Eugenio Garza Sada 2501, Monterrey, Nuevo Leon 64849, Mexico; f Analytical Services International, St George’s University of London, London, United Kingdom; g Environmental Research Group, School of Public Health, Faculty of Medicine, Imperial College London, London, United Kingdom; h School of Sustainable Engineering and the Built Environment, Arizona State University, Tempe, Arizona, USA; i OneWaterOneHealth, Arizona State University Foundation, 1001 S. McAllister Avenue, Tempe, AZ 85287-8101, USA; j AquaVitas, LLC, 9260 E. Raintree Dr., Ste 140, Scottsdale, AZ 85260, USA

**Keywords:** Wastewater, Direct injection LC–MS/MS, Pharmaceuticals, Illicit drugs, Pesticides

## Abstract

A rapid quantitative method for 135 contaminants of emerging concern (CECs) in untreated wastewater enabled with direct injection liquid chromatography-tandem mass spectrometry is presented. All compounds were analysed within 5 min on a short biphenyl cartridge using only 10 μL of filtered sample per injection. Up to 76 compounds were monitored simultaneously during the gradient (including mostly two transitions per compound and stable isotope-labelled analogues) while yielding >10 data points per peak. Evaluation of seven solid phase extraction sorbents showed no advantage for wastewater matrix removal. Excellent linearity, range, accuracy and precision was achieved for most compounds. Matrix effects were <11 % and detection limits were <30 ng L^−1^ on average. Application to untreated wastewater samples from three wastewater treatment works in the UK, USA and Mexico, enabled quantification of 56 compounds. Banned and EU ‘watch-list’ substances are critically discussed, including pesticides, macrolide antibiotics, diclofenac, illicit drugs as well as multiple pharmaceuticals and biocides. This high-throughput method sets a new standard for the speedy and confident determination of over a hundred CECs in wastewater at the part-per-trillion level, as demonstrated by performing over 260 injections per day.

## Highlights

• Rapid, direct LC–MS/MS of 135 analytes in filtered wastewater demonstrated in 5 min.
• Quantitative performance was excellent over several orders of magnitude.
• Median limits of detection of 9 ng L^−1^ with standard injection volumes and flow rates.
• Excellent chromatographic definition with ∼20−30 datapoints/peak.
• 56 compounds quantified in small samples of raw wastewater from UK, USA and Mexico.

## Graphical abstract

**Figure:**
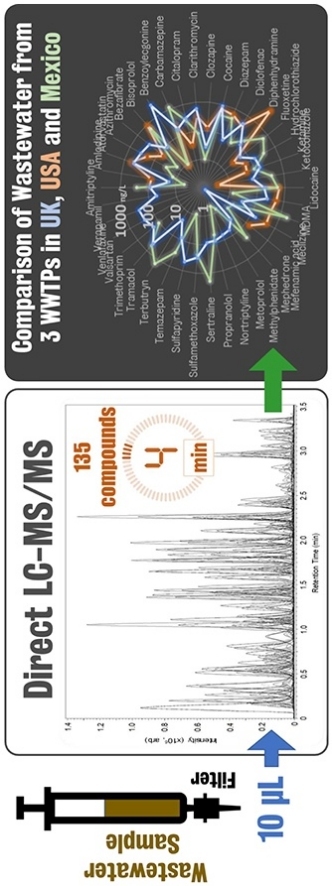

## 1 Introduction

Contaminants of emerging concern (CECs), such as pharmaceuticals, illicit drugs, pesticides, herbicides, personal care products and each of their metabolites/transformation products are being ubiquitously found in a variety of environmental compartments at parts per billion/trillion concentrations given their widespread usage in healthcare, recreational/illicit drug use, and agriculture. Monitoring population-level consumption behaviour and/or exposure to such substances through wastewater-based epidemiology (WBE) has become a viable means to gather near real-time information on temporal and spatial trends across towns and major cities globally for a number of years (Ort et al., 2014; Ryu et al., 2016). Regarding environmental exposure to CECs, wastewater has been identified as a primary source of contamination in receiving waters and soils (Munro et al., 2019; Gibson et al., 2010). This has led to a large body of research focussing on their occurrence, fate and effects in biota and ecology (Kinney et al., 2008; Zenker et al., 2014; Miller et al., 2018) including establishment of an EU ‘watch list’ for CECs (Negrão de Carvalho et al., 2015).

Most analytical techniques for targeted CEC determinations have used liquid chromatography-tandem mass spectrometry (LC–MS/MS) for pharmaceuticals (Paíga et al., 2019; Yuan et al., 2014), illicit drugs (Bannwarth et al., 2019; Centazzo et al., 2019; Baker et al., 2014) and pesticides (Zhang et al., 2011; Jones et al., 2017; Köck-Schulmeyer et al., 2013) in wastewaters. LC–MS/MS using triple quadrupole mass analysers has dominated targeted CEC analysis due to their sensitivity, quantitative precision and selectivity via multiple reaction monitoring (MRM) (Malachová et al., 2018). However, for large numbers of compounds, triple quadrupoles can often be limited by a maximum number of simultaneous MRM transitions which, for hundreds of CECs, can be further constrained by the requirement for multiple transitions per compound for confirmation. This has been generally overcome by scheduling MRMs within defined retention time windows to maximise coverage as well as peak definition and sensitivity, but chromatographic efficiency and resolution also remains important. Therefore, fast-scanning mass analysers are desirable to increase throughput. Analysis of large numbers of compounds using liquid chromatography-high resolution mass spectrometry (LC–HRMS) has also proved effective including the potential flexibility for discovery of new compounds, metabolites and transformation products along with simultaneously performed targeted analysis (Bade et al., 2015; Barron and McEneff, 2016; Munro et al., 2015). For a number of reasons, HRMS detectors are still not achieving the sensitivity of quadrupole-type instruments by comparison (Martínez Bueno et al., 2007; Brown and Wong, 2015). Faster HRMS scan speeds may be required using sub-maximal resolution settings to adequately define narrow chromatographic bands for quantitative applications at ng L^−1^ sensitivity (Munro et al., 2015; Rodriguez-Aller et al., 2013). Aside from LC–MS analysis speed, sample pre-treatment involving solid-phase extraction (SPE) is widely applied in environmental analysis of CECs to achieve sufficient sensitivity at low to mid ng L^-1^ levels (Petrie et al., 2014; Ternes, 2001; Ruff et al., 2015). However, SPE method development for so many compounds is often very complex to optimise and time-consuming, costly and impractical for application in high-volume monitoring campaigns. The large array of chemically diverse compounds and their metabolites makes the availability and selection of suitable sorbents a challenge (Rapp-Wright et al., 2017). Thus, a need for making compromises arises and the SPE process can limit the analytical coverage for complex mixtures.

In comparison to those methods employing SPE, few ‘direct injection’ LC–MS/MS-based methods exist for CECs. Of those that have been developed, most have been developed for small numbers of compounds (Hao et al., 2016; Ramos et al., 2017; Vosough et al., 2015). Among these methods for >20 compounds, for example, large sample injection volumes of 80−400 μL (Anumol et al., 2015; Borrull et al., 2019; Nürenberg et al., 2015) have been used along with relatively long gradients (Reemtsma et al., 2013; Greulich and Alder, 2008), or separate runs for each electrospray ionisation (ESI) source polarity to confidently achieve the robust ng L^−1^ sensitivity required (Oliveira et al., 2015; Hermes et al., 2018). The fastest reported analysis time for larger numbers of CECs in wastewater was reported in 2017 by Campos Mañas et al. as 31 min (Campos-Mañas et al., 2017), using two separate methods and 10 μL injection volumes onto an LC-quadrupole-linear ion trap MS instrument enabling ∼46 injections per day. In many cases CECs are relatively polar molecules and most studies have used C_18_ stationary phases for LC separations. More recently, biphenyl stationary phases have emerged as a potential alternative (Albergamo et al., 2018). Couchman et al. recently configured a short 5 × 3 mm biphenyl guard column directly to the ESI source to perform rapid separations of 20 drugs and metabolites in blood in 36 s using a high mobile phase flow rate of 2 mL min^−1^ (Couchman et al., 2018). The method was then applied to the quantification of clozapine and norclozapine in 76 plasma samples within 3 days (including data processing and interpretation) and lower limits of quantification (LLOQs) lay at 10 ng mL^−1^ in matrix for both analytes. This approach potentially offers several advantages for high-throughput monitoring of mid-polarity CECs in wastewaters. Therefore, even though direct injection-type methods remain rare, the current challenge lies in the speed of LC–MS/MS analysis to improve throughput for large monitoring campaigns at reduced cost while maintaining analytical quality.

The aim of this work was to develop a rapid, direct injection LC–MS/MS methodology for simultaneous quantification of over one hundred selected CECs, including pharmaceuticals, pesticides, illicit drugs and their metabolites at ng L^−1^ concentrations in influent wastewater. Challenges relating to the consolidation of methods using ESI polarity switching, run time, data quality, injection volume and sensitivity were all addressed as a priority. Furthermore, the use of SPE for matrix removal was assessed to determine any sensitivity enhancement. The performance of the method was evaluated with respect to precision, accuracy, matrix effects, linearity, range, limits of detection and quantitation. Lastly, wastewater samples from selected wastewater treatment plants (WWTPs) from the UK, USA and Mexico were analysed using the developed high throughput method. The novelty of this work lies in the improved simplicity and convenience for sample preparation and the successful application of ultra-fast LC–MS/MS transition scanning to enable the determination of 135 compounds for application in WBE, with up to 261 injections performed in any 24 -h time period.

## 2 Materials and methods

### 2.1 Reagents, chemicals and consumables

LC–MS grade methanol (Dorset, UK), LC–MS grade acetonitrile (Rehovot, Israel), hydrochloric acid (37 %, v/v) (Steinheim, Germany), formic acid (Steinheim, Germany) were acquired from Sigma-Aldrich. Ultrapure water (resistance of 18.3 MΩ cm) was generated from a Millipore Milli-Q water purification system (Millipore, Bedford, MA, USA). Calcium chloride diydrate (Acros Organics, Loughborough, UK), magnesium sulfate (Sigma-Aldrich, Steinheim, Germany), potassium chloride (Alfa Aesar, Heysham, UK) and sodium hydrogen carbonate (Fisher Scientific, Loughborough, UK) were used to prepare artificial freshwater at concentrations of at 80, 12, 3 and 17 mg L^−1^, respectively. A list of all 135 reference standard materials and 27 stable isotope labelled internal standards (SIL-IS) is given in the supplementary information. Working standards (either using 1.0 mg mL^−1^ or 0.1 mg mL^−1^ reference standards and as the free base form for HCl salts) were prepared in methanol or acetonitrile and stored in silanised amber vials (20 mL) at −20 °C.

### 2.2 Instrumentation

Liquid chromatography was performed using a Shimadzu Nexera™ X2 ultra-high pressure LC (Shimadzu Corporation, Kyoto, Japan) on a short 5.0 × 3.0 mm, 2.7 μm particle size Raptor™ biphenyl cartridge (Thames Restek, Saunderton, UK) housed within an EXP® Direct Connect Holder. Mass spectrometry was performed using an LCMS-8060 (Shimadzu Corporation, Kyoto, Japan). As the electrospray ionisation (ESI) source was not electrically grounded, the column was configured via a short piece of narrow bore polyether ether ketone (PEEK) tubing. A sample injection volume of 10 μL was used at an optimised flow rate of 0.5 mL min^−1^. Mobile phases were 0.1 % (v/v) formic acid in ultrapure water (A) and 0.1 % (v/v) formic acid in acetonitrile:methanol (1:1, v/v) (B). Optimised gradient elution conditions were as follows: 10 % mobile phase B for 0.2 min; a linear ramp from 10 to 60 % from 0.2 to 3.0 min; a step gradient from 60 to 100 % at 3.0 min; and held at 100 % B for a further 1.0 min before re-equilibration time for 1.0 min, resulting in total run time of 5.0 min. Between runs, a 30 s period was also necessary for needle washing (acetonitrile) and autosampler cycling for the next sample.

For LC–MS/MS, Pureshield argon was used as a collision-induced dissociation (CID) gas (BOC Gases, Guildford, UK). Nitrogen and dry air were generated using Genius 1051 gas generator (Peak Scientific, Inchinnan, UK). Multiple reaction monitoring (MRM) was performed with positive-negative ionisation polarity switching. The quadrupoles Q1 and Q3 were set to unit resolution. Chromatographic data were acquired by LabSolutions™ (version 5.93, Shimadzu) and processed using LabSolutions Insight (version 3.2, Shimadzu, Kyoto, Japan). Automated MRM optimisation of each precursor was performed using LabSolutions software (version 5.93, Shimadzu). All MRM parameters, including product ion *m/z*, collision energy (CE), dwell time, pause time, Q1 and Q3 pre-bias voltages were determined and optimised via 10 μL flow injection LC–MS at ambient temperature without an analytical column. Sample was delivered under isocratic conditions at 70 % mobile phase B and a flow rate of 0.5 mL min^−1^. MRM parameters were optimised using individual analyte standards in methanol at 1.0 μg mL^−1^. Two MRM transitions were used where possible for confirmation of analytes, and the most intense transition used for quantification. For SIL-IS, only one transition was used for quantification purposes. The MS conditions and optimised MRM transitions are summarised in Tables S1 and S2 in the Supplementary Information.

### 2.3 Method validation

The method was validated for the analysis of wastewater samples with direct injection LC–MS/MS according to guidelines published by the International Council for Harmonisation of Technical Requirements for Pharmaceuticals for Human Use (ICH) (Validation of analytical procedures: text and methodology Q2(R1), 2005). Raw wastewater from London was used for analytical performance testing using a pooled mixture of wastewater taken over seven days. Linearity, range, lower limit of detection (LLOD), LLOQ, precision and matrix effects (ME) were assessed as per the guidance. Background subtraction was performed for any analyte already present in the sample as required. Briefly, acceptable linearity and range were defined based on a minimum of N ≥ 5 calibrants yielding coefficients of determination (R^2^) ≥0.99 from a set range of matrix-matched standards tested covering N = 11 concentration levels from 5 to 5000 ng L^−1^. The LLOD was calculated as three times the standard deviation of the response at the lowest calibrant in the defined range. LLOQ was determined as ten times this standard deviation. Precision was performed at 100 and 1000 ng L^−1^ (*n* = 6 at each concentration) in matrix and expressed as percentage relative standard deviation (%RSD). Accuracy of the method was performed at three concentrations levels, i.e., 250 and 750 ng L^−1^ (each in duplicate) and 1000 ng L^−1^ (for *n* = 6). Fortified wastewater was prepared as a quality control (QC) and analyte concentration was determined from the matrix-matched calibration curve and reported as the percentage of coefficient of variation (%CV) difference between the target and QC concentrations, with %CV ≤ ±25 % considered acceptable. ME were determined at 100 and 1000 ng L^−1^ (*n* = 6 at each level) and expressed as a percentage of the peak areas obtained for background subtracted matrix-matched standards relative to those obtained for a standard of all analytes at the same concentrations prepared with ultrapure water.

### 2.4 Sample collection and preparation

A total of 17 samples were taken in three different WWTPs in Monterrey (Mexico), London (UK), and a third city in the USA to demonstrate the feasibility of the approach for large scale international monitoring campaigns in the future. Sites were not selected based on priority, but based on access to samples by the collaborating academic institutions. However, no CEC occurrence data currently exists in the literature for the Monterrey site. Different standard procedures were employed for sample collection at each location. In the UK and the USA, 24 -h 30-min time-proportional composite influent wastewater samples were collected from major metropolitan areas. In London, samples were taken at a major WWTP over a weekend from 5−7 April 2019 (population served by WWTP: 3,400,000 or ∼40 % of Greater London). Each day, 6 × 500 mL sub-samples of the full composite wastewater sample were transferred to Nalgene bottles, which were pre-rinsed with methanol and ultrapure water to avoid potential contamination and shipped at 4 °C to the laboratory. A single 500 mL grab sample of river water (River Thames, UK) was taken in a Nalgene bottle in the same way on 01/07/2019 from Gabriel’s Pier in Central London (51°30′30.3″N; 0°06′36.7″W). UK river and wastewater samples were then filtered using Whatman® 47 mm diameter, 0.67 mm thickness, 2.7 μm pore size GF/D glass microfibre filters (Fisher Scientific Ltd., Loughborough, UK) under vacuum and stored at −20 °C until analysis. These filters were used to minimise analyte losses via sorption. Samples from the USA were collected from the 9–15 September 2019 at an anonymised WWTP in the south-west of the country (population served: 60,888, ∼33 % of the immediate surrounding city area population of 180,000). Upon collection, samples were immediately put on ice and transported to the partnering laboratory in the USA within 24 h. Following this, frozen 5−10 mL aliquots were sent in amber glass vials to London over 24−48 h and stored in the freezer (−20 °C) until analysis within one week. Finally, grab samples of influent wastewater were taken from Mexico for a full week (19–25 February 2019) from Dulces Nombres WWTP (Monterrey) using Nalgene bottles and were acidified to pH 2 using HCl and again 10 mL aliquots were shipped frozen in glass containers to the London laboratory within 24−48 h where they were kept frozen until analysis. This WWTP serves a population of 1,708,190 (∼44 % of the population of the surrounding metropolitan area including the municipalities of San Pedro, Guadalupe, Dulces Nombres, Santa Catarina, Apodaca and part of Monterrey city itself). Given the smaller volumes available for USA and Mexico samples, particulates were removed using single-use 0.2 μm Teflon membrane filters configured to BD Plastipak™ syringes. All samples shipped from overseas were still ice cold upon receipt, which minimised the possibility of analyte loss from degradation (Fedorova et al., 2014). As Monterrey samples were also acidified, this has previously been shown to further improve the stability of pharmaceuticals and illicit drugs in wastewaters (Baker and Kasprzyk-Hordern, 2011). However, to simulate the 48 -h transit period, relative analyte stability was also confirmed. For this, six spiked aliquots of wastewater were prepared at 500 ng L^−1^ (including SIL-IS), not acidified and frozen. Three aliquots were removed and left to thaw on the bench over 48 h with no added cold insulation or ice storage, and then analysed by LC–MS/MS. The relative % instability was calculated using a ratio of the mean peak areas measured in the thawed and frozen wastewater samples, respectively.

### 2.5 Quantification procedures for CECs in influent wastewater

To maintain dilution factors and to prepare matrix-matched calibrants and for fortification with SIL-IS, a fixed volume of 100 μL of standard/SIL-IS standard solutions in methanol was added to 900 μL of filtered wastewater. For quantification of CECs, matrix-matched, background-subtracted calibrations were performed for each WWTP separately via fortification with all analytes over a range of 0–5,000 ng L^−1^ (*N* = 13) along with all 27 SIL-IS at a fixed concentration of 500 ng L^−1^ into a pooled mixture of all samples. For analytes where corresponding SIL-IS were available, quantification was performed using sample peak area ratios relative to those within the background subtracted, matrix-matched calibration curve. For quantification of compounds where no SIL-IS were available, standard addition calibration was performed using their peak areas directly. All statistical analysis was performed in Microsoft® Office Excel (WA, USA).

## 3 Results and discussion

### 3.1 Direct LC–MS/MS method development

For development of a direct LC–MS/MS method for routine wastewater monitoring, several critical issues needed to be considered and resolved first. A relatively rapid separation time was preferred to enable high-throughput and to assess any gains in sensitivity. Secondly, careful scheduling of MRM transitions and MS loop times were necessary to ensure sufficient data acquisition frequency for reliable quantification, ideally as a single run and to include ESI polarity switching. Finally, circumvention of extensive matrix removal procedures or use of large injection volumes to achieve ng L^−1^ sensitivity for real samples were investigated.

The rapid LC–MS/MS approach by (Couchman et al. (2018)) was adapted and further optimised. Initial mobile phase conditions of 10 % B enabled better resolution of more compounds and with better and linear distribution across the runtime. The ratio between mobile phase (via flow rate) and injection volumes was investigated. Gradient events were kept proportional over incremental runtime lengths using 0.1−2 mL min^−1^ flow rates (using a constant 10 μL injection volume). Peak intensities of 27 SIL-IS in influent wastewater from London reached a maximum at 0.5 mL min^−1^ (Fig. S1). For some compounds, a two- to three-fold intensity improvement was achieved (e.g., benzoylecgonine, risperidone and tramadol). At lower flow rates, matrix suppression was most likely the cause of lower intensity (despite a smaller sample dilution factor) rather than excessive band broadening. On the other hand, reduced intensity at higher flow rates were most likely due to excessive dilution of sample. Chromatographic efficiency was also four-fold better at 0.5 mL min^−1^ in comparison to the original 2.0 mL min^−1^ flow used by Couchman et al. (i.e., plate height (HETP) ≈7 μm and number (*n*) ≈135,000 plates/m (Fig. S2) (Couchman et al. (2018)). ‘Dilute-and-shoot’ methods have become popular in recent years, but an offline dilution step was successfully removed as a result of this approach.

**Fig. 1:**
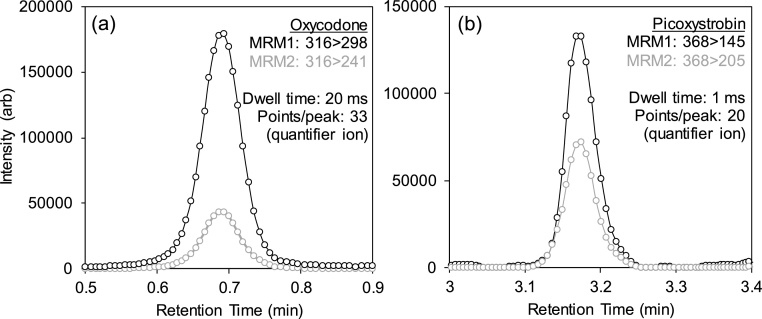
MRM data acquisition frequency and chromatographic peak definition for wastewater spiked with 500 ng L^−1^ of (a) oxycodone (an opioid pharmaceutical) and (b) picoxystrobin (a broad-spectrum fungicide) representing sharper eluting bands of all compounds and measured using two different dwell times of 1 and 20 ms.

The LCMS-8060 instrument has a maximum scan speed of 30,000 u/sec and a polarity switching speed of 5 ms with a capability to acquire 555 MRMs per second. According to the manufacturer, the ion signal response for each MRM is not influenced by the number of other MRM transitions in the same time window. This enabled monitoring of 292 MRM transitions in one run using rapid polarity switching. With a typical peak width of 10−20 s and with dwell times between 1−20 ms, more than 10 data points per peak could be generated (e.g., see Fig. 1 for oxycodone and picoxystrobin). Overall, this level of definition was maintained for up to 76 compounds monitored simultaneously with mostly two MRM transitions per compound in addition to any SIL-IS SRM transitions. With an injection-to-injection time of 5.5 min, up to 261 injections could be performed in a 24 h period which, to our knowledge, represents the highest throughput in this field for monitoring so many CECs in wastewater in a single run with polarity switching enabled.

**Fig. 2:**
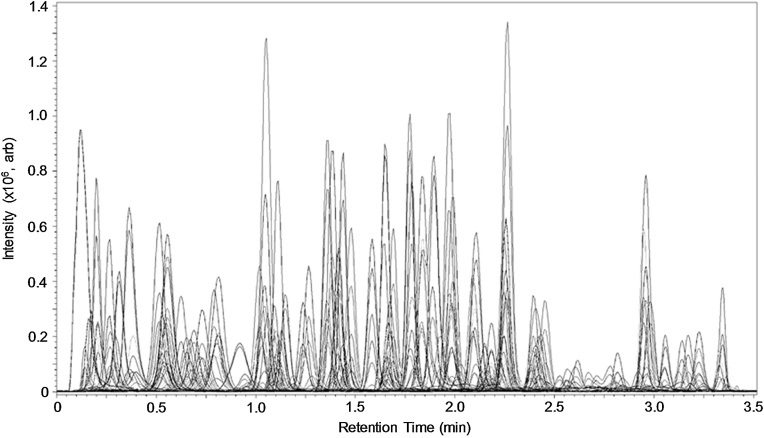
LC–MS/MS chromatogram of a standard mixture containing 135 pharmaceuticals, illicit substances, metabolites, pesticides and 27 SIL-IS.

Using a 500 ng L^−1^ SIL-IS spiked wastewater sample and injection volumes of 0.5−20 μL, it was found that signal intensity deviated from linearity above 10 μL (Fig. S3(a)) and for several compounds peak shape deteriorated. Secondly, and as perhaps expected, the variance in replicate measurements decreased as injection volume increased and %RSDs lay below 5 % on average for 10 and 15 μL injection volumes (Figure S3b). The optimised separation of all compounds and SIL-IS spiked into a London wastewater sample is shown in Fig. 2. The sensitivity of the method was considered suitable for direct analysis at this point, but obviously could be improved using analyte-selective SPE for enrichment, but would add considerable time. Alternatively, SPE was considered here for active matrix removal as a more practically convenient way to improve sensitivity and increase throughput (i.e., by minimising any extra time, as analytes were collected in the SPE eluate after loading). Single or combinations of sorbents with little/no analyte recovery could prove beneficial to minimise ME, as employed recently for trace explosives determination in wastewater (Irlam et al., 2019). This was evaluated using two matrices, filtered artificial freshwater and raw wastewater (each spiked at 500 ng L^−1^ with a selection of 105 analytes that were in stock at the time). Both types of sample were analysed directly by the optimised LC–MS/MS method and compared to extracts of corresponding samples that were subject to SPE with no prior pH adjustment. The resulting peak areas were expressed as a percentage and shown in Table S3. In general, peak areas were much lower for most compounds in samples subjected to SPE and some were not detected at all. It was concluded that samples should be analysed directly following filtration only.

### 3.2 Direct LC–MS/MS method performance for CECs in influent wastewater

**Fig. 3:**
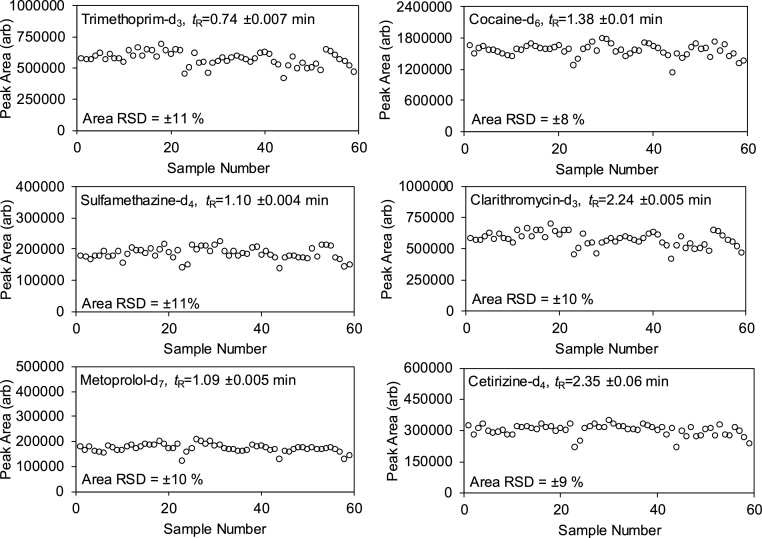
Peak area and retention time stability for selected SIL-IS over a sequence of *n* = 59 spiked London wastewater samples (500 ng L^−1^) and measured using direct LC–MS/MS analysis over a total batch analysis time of 6.4 h.

A summary of method performance for all 135 CECs determined in London influent wastewater is shown in Table 1 (full data for each compound in Table S4). Linearity was excellent for most compounds with coefficients of determination of R^2^ ≥0.99 for 127 (94 %) compounds. Limited sensitivity was the general cause for poorer performance for the eight remaining compounds and especially for cymoxanil, norethisterone, prodiamine and indomethacin where R^2^ was ≥0.99, but for *n*<5 calibrants at the higher concentration range. Overall, the imprecision in peak area (expressed as mean (±standard deviation)) was excellent at 11 (±10) % and 8 (±6) % on average at 100 and 1000 ng L^−1^, respectively. Over 82 % of compounds displayed %CV ≤ 15 % at both concentrations. The highest variance was noted for diflubenzuron and prodiamine at both concentration levels (52 and 32 % RSD, respectively). Precision over a sequence of *n* = 59 spiked wastewater samples was also assessed using SIL-IS internal standards at 500 ng L^−1^ in wastewater (see Fig. 3 for a selection). In general, there were no major drifts or deviations in either retention time or peak area. It is highly likely that the low injection volume contributed to high stability in chromatographic performance and mass spectrometry response though some evidence of matrix deposition within the ion source at the end of long batch sequences was observed (Fig. S4). No reduction in LC–MS/MS performance was evident throughout this study. Lastly, mean (±standard deviation) accuracy at 250, 750 and 1000 ng L^−1^ lay at −13 (±17) %, −8 (±9) % and −6 (±10) % respectively, which was also considered acceptable.

**Table 1:** Summary of analytical performance characteristics for all 135 CECs using direct injection LC–MS/MS. For full individual analyte data, please refer to Table S4.

	Linearity	Peak Area Precision		Matrix Effect		Inaccuracy			Sensitivity	
	*N*≥5 (max *N* = 12)	RSD%, *n* = 6		CV%, *n* = 6		CV%^a^			LLOD^b^	LLOQ^c^
	R^2^	at 100 ng L^−1^	1000 ng L^−1^	at 100 ng L^−1^	1000 ng L^−1^	250 ng L^−1^	750 ng L^−1^	1000 ng L^−1^	ng L^−1^	ng L^−1^
Maximum	0.999	55	32	+337	+188	+66	+13	+9	533	1777
Minimum	0.967	2	1	−84	−60	−97	−54	−44	0.06	0.21
Absolute Median	0.999	8	6	11	9	12	8	−4	9	31
Absolute Mean (± standard deviation)	0.998 (±0.0037)	11 (±10)	8 (±6)	20 (±34)	14 (±22)	16 (±14)	9 (±7)	−6 (±10)	29 (±59)	95 (±197)

^a^for each of 250 and 750 ng L^−1^ levels, accuracy represents the mean of two replicate matrix-matched standards, for 1000 ng L^−1^ it represents the mean of n = 6 replicates.

^b^Lower limit of detection.

^c^Lower limit of quantitation.

Sensitivity was excellent for such a simple analytical method. LLODs varied from 0.05 (for memantine) to 533 ng L^−1^ (for carfentrazone-ethyl). The median LLOD and LLOQ were determined at 9 and 31 ng L^−1^, respectively (average LLOD = 29 ng L^−1^). In comparison to other direct LC–MS/MS methods for influent wastewater, this method displayed largely similar or better sensitivity in some cases though there were relatively few common compounds for a full comparison (and especially when injected analyte mass on column is considered). However, for at least two previously published methods (Oliveira et al., 2015; Hermes et al., 2018), this method used five to ten-fold smaller injection volumes which could reduce the amount of matrix contamination of the ESI source over longer batch analyses. The remaining method by Campos-Mañas et al. also used 10 μL injection volumes (Campos-Mañas et al., 2017), but with two separate longer gradient runs (total analysis time 31 min). On average, MEs for all 135 compounds spiked at 100 and 1000 ng L^−1^ in wastewater were −3 (±40) % and 0 (±26) %. However, by taking the absolute value of % suppression (−) or enhancement (+) data, the calculated overall median was 11 % ME for all compounds, again showing excellent performance. It was noted that the highest MEs were observed for antipyrine (−84 %, indicating enhancement) and spiramycin (+337 %, indicating suppression) at 100 ng L^−1^ spiking concentration and for clodinafop-propargyl (−60 %) and spiramycin (+188 %) at 1000 ng L^−1^. The relative absolute mean instability of analytes in spiked wastewater samples measured after thawing frozen spiked samples over 48 h was 7 (±12) % (n = 3, Table S5) and not considered significant for most analytes. However, instability was particularly high for azelnidipine, ketoconazole and fenoxaprop-ethyl with +85, +73 and +58 % loss, respectively, which indicated either that the change in matrix led to a suppression in signal, or that these compounds transformed rapidly over this time, For compounds with increased signal in thawing samples, transformation of other related substances present in the sample could have led to this result (e.g., cleavage of conjugated metabolites) or the variance across replicate samples was higher. As quantification for all sites was performed using matrix-matched standards prepared at the same time, much of the suppression component of this apparent difference was likely to have been accounted for. However, reported concentrations of these compounds in wastewater samples should be treated with caution, as it was impossible to accurately account for stability in every sample received.

### 3.3 Analysis of wastewater samples from the UK, USA and Mexico

A total of 58 individual compounds were detected across all samples and, of these, 56 were quantifiable (Table 2). No carryover was observed between matrix-matched calibrants, standards, blanks and/or samples. The approximate percentage of the national population covered by these works in each country was UK = 5 %, Mexico = 2 % and USA < 1 %. Therefore, extrapolation to perform international comparisons on this level was not appropriate. Our primary focus was therefore placed on a catchment level comparison in this preliminary study using the new direct analysis method, which conveniently enabled shipment of several small samples internationally to be analysed in one laboratory under the same conditions.

**Table 2:** Occurrence of CECs in influent wastewater samples from three WWTPs from the UK, Mexico and the USA measured using direct LC–MS/MS analysis (average of n = 3 replicates ± standard deviation).

Analyte	London, UK (5−7^th^ April, 2019) WWTP Population: 3.4 M			Monterrey, Mexico (19^th^-25^th^ Feb, 2019) WWTP Population: 1,708,190							WWTP in Southwestern USA (9^th^-15^th^ Sept., 2019) WWTP Population: 60,888 (as 24-h composite samples)						
	(as 24-h composite samples)			(as grab samples)													
	Sat	Sun	Mon	Tues	Wed	Thu	Fri	Sat	Sun	Mon	Mon	Tue	Wed	Thu	Fri	Sat	Sun
4-Methyl-ethcathinone	–	–	–	–	–	–	913 ± 12	–	–	–	–	–	–	–	–	–	–
Acetamiprid	–	–	–	–	–	34 ± 6	–	–	–	–	–	–	–	–	–	–	–
Ametryn	–	–	–	–	–	–	–	99 ± 4	–	–	–	–	–	–	–	–	–
Amitriptyline	95 ± 9	72 ± 4	79 ± 11	–	–	–	–	–	–	–	72 ± 5	77 ± 4	82 ± 5	87 ± 7	81 ± 4	78 ± 2	75 ± 4
Amlodipine	30 ± 12	10 ± 14	12 ± 10	113 ± 11	–	–	–	112 ± 21	–	–	–	–	–	–	–	–	–
Antipyrine	<LLOQ	–	<LLOQ	–	–	–	–	–	–	–	–	–	–	–	–	–	–
Atorvastatin	446 ± 25	414 ± 27	485 ± 10	–	–	–	–	–	–	–	<LLOQ	<LLOQ	<LLOQ	<LLOQ	<LLOQ	<LLOQ	–
Atrazine	–	–	–	–	–	–	–	48 ± 3	35 ± 5	26 ± 1	–	–	–	–	–	–	–
Azithromycin	324 ± 71	356 ± 99	386 ± 26	<LLOQ	<LLOQ	<LLOQ	<LLOQ	<LLOQ	<LLOQ	<LLOQ	391 ± 35	410 ± 49	545 ± 27	865 ± 79	721 ± 105	499 ± 53	403 ± 47
Azoxystrobin	–	–	–	–	–	–	–	–	–	–	321 ± 5	127 ± 3	189 ± 8	207 ± 10	212 ± 12	169 ± 9	137 ± 4
Bezafibrate	263 ± 20	290 ± 24	307 ± 24	<LLOQ	<LLOQ	<LLOQ	<LLOQ	<LLOQ	4375 ± 136	<LLOQ	–	–	<LLOQ	<LLOQ	<LLOQ	–	–
Bisoprolol	77 ± 7	83 ± 2	83 ± 5	10 ± 1	9 ± 1	8 ± 2	11 ± 2	12 ± 2	9 ± 2	8 ± 1	–	–	–	–	–	–	–
Bupropion	–	–	–	–	–	–	–	–	–	–	<LLOQ	<LLOQ	23 ± 7	40 ± 7	162 ± 7	160 ± 13	71 ± 12
Benzoylecgonine	2635 ± 376	2786 ± 7	2931 ± 88	998 ± 36	784 ± 21	791 ± 34	949 ± 30	1768 ± 42	1597 ± 119	1196 ± 42	341 ± 9	613 ± 13	754 ± 19	915 ± 42	1263 ± 39	1485 ± 41	988 ± 24
Carbamazepine	30 ± 9	195 ± 14	310 ± 14	290 ± 20	244 ± 17	229 ± 14	276 ± 37	274 ± 39	261 ± 5	223 ± 16	–	–	–	33 ± 4	24 ± 7	–	–
Carbamazepine epoxide	<LLOQ	<LLOQ	<LLOQ	97 ± 3	98 ± 6	95 ± 5	74 ± 8	119 ± 12	–	81 ± 9	–	–	–	–	–	–	–
Citalopram	325 ± 22	303 ± 16	327 ± 11	–	–	–	–	–	–	–	179 ± 14	259 ± 22	270 ± 14	294 ± 6	257 ± 15	216 ± 5	191 ± 1
Clarithromycin	673 ± 68	568 ± 14	536 ± 28	–	–	–	–	–	–	–	244 ± 26	–	–	<LLOQ	<LLOQ	–	–
Clopidogrel	<LLOQ	<LLOQ	<LLOQ	–	–	–	–	–	–	–	–	–	–	–	–	–	–
Clozapine	29 ± 6	24 ± 4	27 ± 2	22 ± 6	6 ± 2	8 ± 3	11 ± 1	10 ± 3	4 ± 2	10 ± 5	–	–	–	–	–	–	–
Cocaine	801 ± 92	660 ± 33	1138 ± 71	296 ± 18	301 ± 19	334 ± 8	358 ± 11	501 ± 8	701 ± 45	402 ± 26	32 ± 2	31 ± 4	36 ± 5	32 ± 6	719 ± 53	1003 ± 87	443 ± 41
Diazepam	69 ± 5	68 ± 3	65 ± 8	–	–	–	–	–	–	–	–	–	–	–	–	–	–
Diclofenac	458 ± 23	521 ± 92	467 ± 46	412 ± 17	341 ± 6	355 ± 39	632 ± 72	453 ± 35	542 ± 42	338 ± 24	139 ± 9	106 ± 11	140 ± 12	105 ± 7	144 ± 21	104 ± 9	143 ± 11
Diphenhydramine	86 ± 15	98 ± 15	139 ± 16	119 ± 4	59 ± 1	54 ± 3	72 ± 4	97 ± 5	72 ± 2	59 ± 10	647 ± 40	713 ± 48	844 ± 8	873 ± 64	682 ± 56	588 ± 34	451 ± 16
Fenuron	–	–	–	190 ± 6	174 ± 4	237 ± 27	123 ± 6	172 ± 14	144 ± 4	156 ± 6	–	–	–	–	–	–	–
Fluoxetine	56 ± 4	50 ± 4	58 ± 7	–	–	–	–	–	–	–	–	–	–	–	–	–	–
Hydrochlorothiazide	133 ± 20	144 ± 22	154 ± 19	826 ± 83	589 ± 42	716 ± 198	581 ± 113	580 ± 32	546 ± 103	597 ± 167	634 ± 58	491 ± 31	645 ± 140	650 ± 111	641 ± 63	719 ± 119	370 ± 81
Ketamine	150 ± 28	160 ± 8	173 ± 7	–	–	–	–	–	–	–	–	–	–	–	–	–	–
Ketoconazole^b^	<LLOQ	<LLOQ	213 ± 26	692 ± 101	399 ± 49	359 ± 53	326 ± 43	361 ± 30	–	236 ± 71	–	–	–	–	–	–	–
Levamisole	–	–	–	–	135 ± 18	–	171 ± 7	207 ± 13	176 ± 32	–	–	–	–	–	–	–	–
Lidocaine	191 ± 25	173 ± 6	167 ± 5	415 ± 15	268 ± 17	385 ± 12	275 ± 19	563 ± 7	300 ± 11	236 ± 12	170 ± 6	318 ± 3	359 ± 4	399 ± 6	560 ± 12	552 ± 4	359 ± 3
Lincomycin	–	–	–	669 ± 32	510 ± 43	533 ± 17	775 ± 48	331 ± 94	488 ± 6	487 ± 37	–	–	–	–	–	–	–
MDMA	140 ± 19	245 ± 12	342 ± 22	–	–	–	–	–	–	–	–	–	–	–	–	–	–
Meclizine	32 ± 3	33 ± 3	33 ± 1	26 ± 11	12 ± 2	16 ± 5	13 ± 5	<LLOQ	–	27 ± 6	–	–	–	–	–	–	–
Mefenamic acid	137 ± 23	162 ± 16	166 ± 28	–	–	–	–	–	–	–	–	–	–	–	–	–	–
Mephedrone	–	–	4 ± 3	–	–	–	–	–	–	–	–	–	–	–	–	–	–
Methamphetamine	–	–	–	1549 ± 16	1714 ± 6	1676 ± 19	1619 ± 20	1713 ± 34	2094 ± 43	1969 ± 21	3405 ± 79	3995 ± 58	5023 ± 33	4845 ± 32	5173 ± 122	4873 ± 81	4269 ± 30
Methedrone	–	–	–	–	–	–	–	127 ± 36	–	–	–	–	–	–	–	–	–
Methylphenidate	–	50 ± 2	48 ± 1	13 ± 0.2	13 ± 1	12 ± 1	15 ± 1	16 ± 1	17 ± 1	13 ± 2	–	–	–	–	–	–	–
Metoprolol	60 ± 1	57 ± 1	60 ± 4	275 ± 8	213 ± 9	226 ± 13	226 ± 9	259 ± 29	209 ± 10	221 ± 11	7 ± 1	–	83 ± 19	47 ± 7	43 ± 17	6 ± 12	–
Nortriptyline	65 ± 2	64 ± 1	67 ± 4	–	–	–	–	–	–	–	–	–	–	–	–	–	–
Orphenadrine	–	–	–	27 ± 1	–	–	–	27 ± 2	–	–	–	–	–	–	–	–	–
Oxazepam	–	–	–	–	–	–	–	–	–	–	75 ± 24	82 ± 18	75 ± 4	<LLOQ	76 ± 8	99 ± 10	<LLOQ
Oxycodone	–	–	–	–	–	–	–	–	–	–	42 ± 3	39 ± 5	50 ± 3	56 ± 4	64 ± 2	67 ± 4	33 ± 3
Prometryn	–	–	–	–	–	–	–	36 ± 2	–	–							
Prometon	–	–	–	–	–	–	–	–	–	–	5 ± 2	4 ± 0.5	3 ± 1	4 ± 3	3 ± 2	4 ± 0.4	3 ± 1
Propranolol	100 ± 5	71 ± 8	72 ± 14	<LLOQ	<LLOQ	<LLOQ	–	<LLOQ	<LLOQ	<LLOQ	–	–	–	–	–	–	–
Sertraline	93 ± 18	74 ± 5	92 ± 5	93 ± 8	69 ± 7	65 ± 5	71 ± 6	64 ± 8	–	–	–	–	–	–	–	–	–
Sulfamethoxazole	317 ± 31	318 ± 107	235 ± 74	2802 ± 107	2938 ± 52	2254 ± 155	882 ± 58	1781 ± 87	<LLOQ	2550 ± 14	446 ± 24	576 ± 49	841 ± 24	769 ± 46	717 ± 15	541 ± 82	496 ± 15
Sulfapyridine	458 ± 47	513 ± 20	449 ± 71	342 ± 15	422 ± 19	539 ± 32	<LLOQ	<LLOQ	<LLOQ	296 ± 11	<LLOQ	<LLOQ	<LLOQ	<LLOQ	<LLOQ	<LLOQ	<LLOQ
Sulfathiazole	–	–	–	63 ± 7	–	–	–	–	–	–	–	–	–	–	–	–	–
Temazepam	80 ± 3	75 ± 3	88 ± 4	–	–	–	–	–	–	–	–	–	–	–	–	–	–
Terbutryn	25 ± 1	23 ± 1	19 ± 2	–	–	–	–	–	–	–	–	–	–	–	–	–	–
Tramadol	512 ± 87	428 ± 7	431 ± 11	309 ± 5	271 ± 14	232 ± 7	272 ± 16	502 ± 11	273 ± 17	248 ± 11	2731 ± 64	243 ± 14	202 ± 13	147 ± 15	183 ± 6	112 ± 6	98 ± 8
Trimethoprim	193 ± 11	147 ± 4	185 ± 15	1741 ± 236	1500 ± 68	1353 ± 77	1579 ± 150	1841 ± 101	1145 ± 5	1337 ± 134	223 ± 13	361 ± 4	580 ± 30	417 ± 5	569 ± 13	401 ± 31	354 ± 8
Valsartan	<LLOQ	341 ± 46	389 ± 24	827 ± 36	428 ± 94	457 ± 87	663 ± 39	1032 ± 25	–	469 ± 61	642 ± 78	411 ± 18	609 ± 67	576 ± 72	477 ± 49	351 ± 33	<LLOQ
Venlafaxine	289 ± 30	256 ± 9	282 ± 23	113 ± 2	96 ± 3	102 ± 4	100 ± 3	120 ± 5	103 ± 5	104 ± 1	52 ± 7	144 ± 16	162 ± 17	208 ± 39	193 ± 16	161 ± 11	69 ± 9
Verapamil	51 ± 3	50 ± 1	51 ± 1	–	–	–	–	–	–	–	–	–	–	–	–	–	–

^a^Concentration determined by extrapolation of the upper range of the matrix matched calibration curve 0–5,000 ng L^−1^ (*n* = 13).

^b^Relative instability of this compound was higher at 73 % on average during stability testing and the reported concentrations here have not taken this into account.

- Denotes not detected (<LLOD).

#### 3.3.1 London, UK

**Fig. 4:**
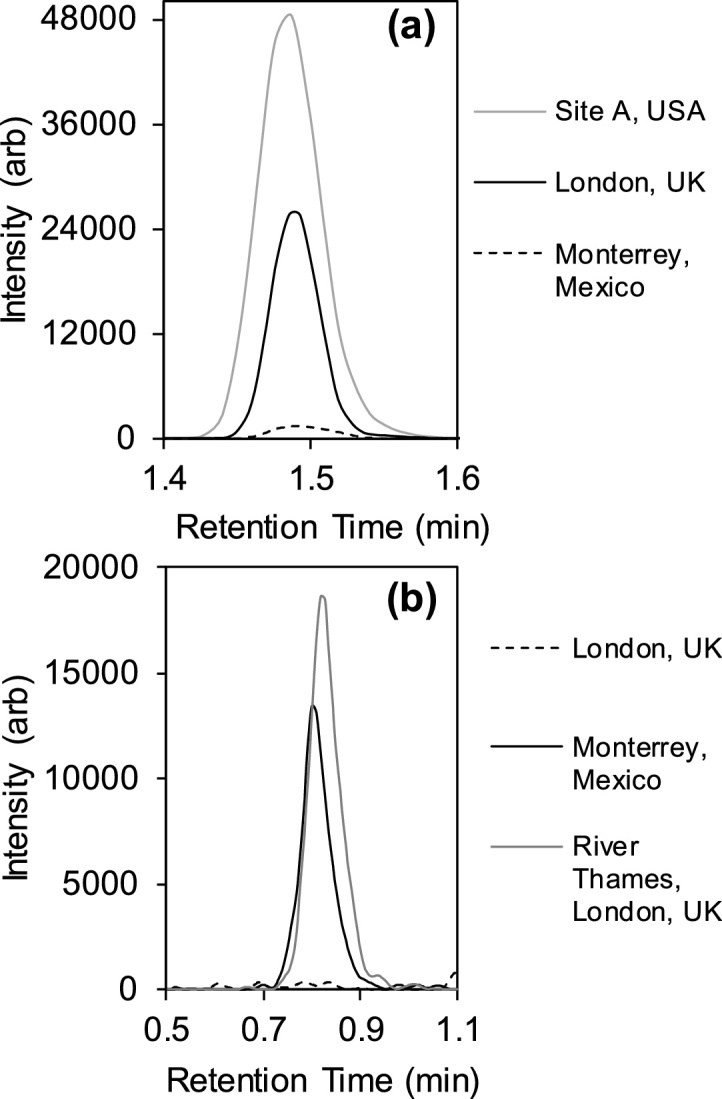
Example SRM transitions using direct LC–MS/MS analysis of influent wastewater from the UK (London), Mexico (Monterrey) and USA showing contamination with (a) azithromycin (London = 324 ng L^−1^, USA = 499 ng L^−1^ and Mexico =<LLOQ) and (b) fenuron (Mexico = 123 ng L^−1^; London = not detected; and Thames river water from Central London = 169 ng L^−1^).

For London wastewater samples, 40–42 compounds were detected each day and quantified concentrations agreed in the main with previous screening work in 2014 using SPE and LC–HRMS) (Munro et al., 2019, 2015). However, this direct LC–MS/MS method included several new compounds, most notably biocides, of which only terbutryn was found in London wastewater samples. Recently, fenuron was determined at high frequency in biota and river water in Suffolk, UK by our group, even though it has been removed from use in the UK (Miller et al., 2019). Fenuron was not detected in London wastewater on this occasion. However, following a preliminary analysis of a Thames River water grab sample taken on the 1st July 2019, fenuron occurrence was again confirmed and following quantification using standard addition calibration (*n* = 12, R^2^ = 0.994), it was quantified at 169 (±5) ng L^−1^ (Fig. 4b). Therefore, given the LLOQ for this compound in influent (50 ng L^−1^), treated wastewater discharged by the London WWTP may not represent a continuous primary source of fenuron to the receiving aquatic environment, but more spatial and temporal monitoring is required to locate its source(s). Aside from pesticides, relatively little recent occurrence data exist for EU ‘watch-list’ compounds present in influent wastewater from Central London including diclofenac, clarithromycin and azithromycin (Fig. 4a) which were all determined at mean concentrations of 482 (±34), 592 (±72) and 355 (±31) ng L^−1^, respectively, across all three days. This represented approximately 1.5-fold the average concentrations determined for each compound in influent at five WWTPs upstream from London which also discharge into the Thames River and as reported recently by (Nakada et al. (2017)). In the Thames River grab sample, 117 (±18) ng L^−1^ and 31 (±10) ng L^−1^ were determined for diclofenac and clarithromycin, respectively (no azithromycin was detected).

**Fig. 5:**
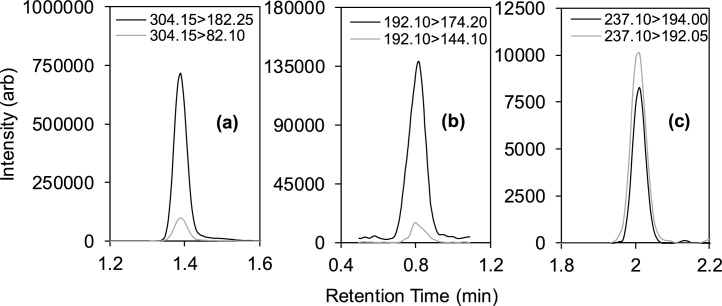
Example MRM chromatograms for selected analytes detected in wastewater samples from June 2019 including (a) cocaine (801 ng L^−1^, London), (b) 4-MEC (913 ± 12 ng L^−1^, Mexico) and (c) carbamazepine (30 ng L^−1^, London).

In addition to pharmaceutical compounds, our group has also contributed illicit drug monitoring data for London wastewater from 2011−2019 as part of several international WBE studies. Validated methods at each laboratory are normally subject to annual international laboratory scrutiny via blind testing exercises, including the method developed herein for the 2019 campaign, which passed with a threshold Z-Score of <2 (van Nuijs et al., 2018). In previous data, BZE loads in wastewater were seen to rise by approximately two-fold between 2011−2015 to ∼1100 mg/1000 people/day at weekends. Both cocaine and BZE concentrations were measurable in wastewater here for 2019 samples (Fig. 5a), but were slightly lower than those in 2016 (maximum weekend concentrations for cocaine and BZE were 1434 and 3533 ng L^−1^, respectively, in 2016). Taking into account the population served by the WWTP, the daily flow and exfiltration (Castiglioni et al., 2013a), weekend BZE loads for the catchment corresponded to a mean (± standard deviation) of 1015 (±38) mg/1000 people/day, which was similar to weekend BZE loads measured in 2016 (999 mg/1000 people/day). Therefore, this work provides some preliminary evidence that cocaine consumption in London may have plateaued. Conversion of BZE loads to actual cocaine consumed in the catchment using a conversion factor of 3.59 (to take into account the urinary excretion rate of cocaine for different dosages and administration routes (Castiglioni et al., 2013a)) resulted in a mean weekend (Saturday–Monday) cocaine consumption of 3640 (±140 mg)/1000 people/day (all consumption data from here onward are rounded to nearest ten). It is important to note that population estimates are likely to be one of the largest sources of uncertainty for WBE (Jones et al., 2014). For example, the population of Greater London was 8,173,941 people as of the 2011 census. London’s population is expected to be larger now and the movement of people is also not accounted for (e.g., commuting to/from the city for work, tourism and large scheduled events). However, by removing the population from the equation and by multiplying the daily BZE wastewater load by the correction factor for cocaine, a generalised estimate for this catchment was calculated at 12.4 (±0.5) kg/day consumed over this weekend in 2019. This catchment represents only 43 % of the total population of Greater London and therefore the combined consumption in kg/day is likely to be much larger for the whole city. Furthermore, these estimates represent consumption of pure cocaine only and street-level cocaine is likely to be mixed with adulterants and diluents to varying degrees (such as lidocaine, which was also determined here at an average concentration of 177 (±13) ng L^−1^). Therefore, this approach may be useful for government and law enforcement agencies to monitor illicit drug markets in near real-time by covering large numbers of catchments simultaneously. For example, a national wastewater programme has been in effect in Australia since 2016, and such activities may benefit from higher throughput and more comprehensive analytical methods like the one developed herein (O’Brien et al., 2019).

Other illicit drugs unique to wastewater samples from London in comparison to the other two sites studied were ketamine, MDMA and mephedrone, the latter of which was only quantifiable near the LLOQ on the Sunday (which likely represents occurrence due to excretion following Saturday night activity). Mephedrone was last determined by our group in London wastewater in March 2014 between 42 and 160 ng L^−1^ across the week and this indicated significant reduction in population-level consumption following its legal restriction (Munro et al., 2015). For MDMA, the average weekend wastewater load was 88 (±35) mg/1000 people/day. Following this, and by using a correction factor of 4.4 to back-calculate to consumed quantities (Gracia-Lor et al., 2016), MDMA consumption was estimated at 390 (±160) mg/1000 people/day over these three days.

#### 3.3.2 Monterrey, Mexico

Between 24 and 35 compounds were detected each day across the week in Monterrey wastewater. The highest concentrations and occurrence frequency were observed on average for two antibiotics, trimethoprim and sulfamethoxazole at 1499 (±243) and 2201 (±768) ng L^−1^, respectively. Azithromycin was detected every day (Fig. 4(a)), but < LLOQ and lower than either London or USA samples. No clarithromycin was detected. In addition to these antibiotics, lincomycin, sulfapyridine were also quantifiable every day. Very little occurrence data exists for pharmaceuticals in untreated wastewaters from Mexico for comparison and this represents one of the most comprehensive analyses to date. That said, using a LC–MS/MS method for 35 pharmaceuticals, Rivera-Jaimesa et al., quantified 11 compounds in wastewater from Cuernavaca, including the same two antibiotics albeit at lower concentrations of 125−790 ng L^−1^ for trimethoprim and 775−2010 ng L^−1^ for sulfamethoxazole (Rivera-Jaimes et al., 2018). However, four to five-fold higher concentrations of diclofenac on average were observed in Cuernavaca wastewater in comparison to those measured in this study. With respect to the capital, Mexico City, concentrations of up to 320, 450, 2600, 500 and 100 ng L^−1^ for trimethoprim, clarithromycin, metoprolol, diclofenac and bezafibrate, respectively, were recently reported by (Siemens et al. (2008)). Fenuron was also determined in Monterrey wastewater here at consistent concentrations on average across the week at 170 (±36) ng L^−1^. However, wastewater entering this particular WWTP derives mainly from households and a single defined source of fenuron is unclear. It could arise from exposed fruits and vegetables consumed by the population (Devault et al., 2018) either by direct application of pesticides to crops or indirectly via wastewater irrigation, both of which are common practices in Mexico (Friedel et al., 2000; Pérez et al., 2013). According to the European Chemicals Agency, there may also be a contribution from other sources as it is widely used in a number of materials including adhesives, sealants, coating products, polymers, and paints, and for building purposes in fabricated metal products, plastics and electronic goods (European Chemical Agency, 2020).

In comparison to London, concentrations of illicit drugs and of BZE in Monterrey wastewater in particular were less than half on average at 1154 (±390) ng L^−1^. Recreational usage was evident with a two-fold increase in its concentration observed at the weekend. A similar pattern was observed for cocaine across the week and the ratio between both compounds at both sites were also relatively consistent at 0.31 (±0.08) (London) and 0.36 (±0.06) (Monterrey). Unfortunately, however, as composite samplers were not available at this site, reliable back-calculation to determine daily BZE loads from grab samples was not possible for Monterrey to compare per capita usage. In addition to cocaine, other substances were determined including methamphetamine and methedrone. A single water-loss transition (192 > 174) peak was also observed for 4-methylethcathinone (4-MEC) in six out of seven samples. However, as isomers of 4-MEC exist (e.g., 2- and 3-MEC, 3-,4-methylbuphedrone and 2-,3-,4-ethyl methcathinone), its identity could not be confirmed in these samples with a second transition, and especially in the absence of reference material measurements for these other isomers. This single transition for 4-MEC was also detected in all London and USA wastewater samples. One sample from Monterrey yielded two transitions for 4-MEC and its concentration was then determined at 913 ng L^−1^ (Fig. 5(b)). Very few occurrences of 4-MEC have been reported except for Gonzalez-Marino et al. who reported 4-MEC in wastewater from Milan and south western UK at 0.9 (±3.1) and 1.2 (±1.9) ng L^−1^ which was significantly lower than that measured in this study (González-Mariño et al., 2016). Methedrone was determined at comparatively higher concentrations on the Saturday in Monterrey samples. In contrast to London, methamphetamine was determined with consistency every day at 1762 ± 170 ng L^−1^ in Monterrey wastewater with only a marginal (∼15 %) rise in concentration at the weekend potentially, indicating sustained use by the population. Interestingly, MDMA was not detected in Monterrey or any USA samples, again in contrast to London.

Other compounds detected that are worthy of note were clozapine, carbamazepine (CBZ) (Fig. 5(c)) and its metabolite carbamazepine-1011-epoxide which could each be quantified in Monterrey wastewater every day at higher concentrations than observed in London. Carbamazepine is widely used in the treatment of epilepsy, psychiatric conditions, bipolar disorder and is used to treat chronic neuropathic pain (Wiffen et al., 2011). Cytochrome P-450 3A4 is primarily responsible for transformation into its epoxide metabolite and only ∼1% is excreted as CBZ itself in urine (Kerr et al., 1994; Tolou-Ghamari et al., 2013). At therapeutic doses, the epoxide concentration is generally about 20 % of CBZ. Over 90 % of the epoxide is further hydrated to trans-10,11-dihydroxy-10,11-dihydro-carbamazepine before excretion in urine (Russell et al., 2015; Bertilsson and Tomson, 1986) and this metabolite has been detected at higher concentrations than CBZ in wastewater previously (Kaiser et al., 2014). However, the ratio of CBZ to the epoxide in wastewater was higher than expected at ∼35 (±11) % across all samples. Clozapine is used to treat antisocial personality disorder in adults, and it is a gold standard to treat resistant schizophrenia and bipolar disorder. In Mexico, the prevalence of psychiatric disorders has been reported as 6–16 % for males and 2–9 % for females. In children and adolescents, the prevalence is 2–10 % (Medina-Mora et al., 2003; Juárez-Treviño et al., 2019). Clozapine is also an antipsychotic drug and was introduced in Mexico in 1994. In general, however, fewer antipsychotic and antidepressant-type residues were detected in Monterrey wastewater in comparison to London.

#### 3.3.3 WWTP site in Southwestern USA

Comparatively fewer compounds (*n* = 25–27) were detected in wastewater samples from this site. This WWTP serves a smaller and more suburban population in comparison to the other two sites, but still derives from a major metropolitan area. A few occurrences are worthy of discussion. With respect to illicit drugs, methamphetamine was present in all samples at two to three-fold the concentrations of Monterrey (weekly average: 4512 ± 644 ng L^−1^). Like Monterrey, chronic occurrence was observed, but with increased concentrations on weekdays instead. The advantage of composite sampling used at this site allowed more reliable back-calculation to determine community consumption trends across the week. In terms of wastewater loading, methamphetamine was estimated at 1331 ± 167 mg/1000/people per day which exceeds the highest load determined during the 2018 SCORE EU monitoring campaign (Erfurt, Germany, at 211 mg/1000 people/day) (Sewage analysis CORE Group (SCORE), 2019). However, such estimates may need to be treated with caution as sources of methamphetamine in wastewater can also derive from manufacturing activity, which could be significant depending on its scale within a catchment (Gao et al., 2018). Enantiomeric profiling for chiral drugs like methamphetamine has been used to differentiate drug manufacturing effluent from consumption behaviour (Xu et al., 2017; Castrignanò et al., 2018), but unfortunately this was not possible to determine here using this method and lay beyond the scope of this work. Nevertheless, the USA has reported significant methamphetamine misuse for many years with >14.5 M people above the age of 12 (>5% of total population) reported in 2016 as having tried the drug at least once in their lifetime (Substance Abuse and Mental Health Services Administration, 2017). Moreover, ∼1.4 M reported using the drug in the year preceding this survey. Using a back-calculation correction factor of 2.44 (Gracia-Lor et al., 2016), this yielded an average methamphetamine consumption of 3250 ± 410 mg/1000 people/day in this wastewater catchment (equivalent to ∼65 doses/1000 people/day (González-Mariño et al., 2016)). Cocaine and BZE concentrations on the other hand were much lower (328 ± 402 and 908 ± 387 ng L^-1^ on average, respectively) than London and Monterrey, but peaked at the weekend, as expected. However when using 24-h composite samples, the increased concentration observed on this particular Saturday is also likely to include contributions from excretion of unmetabolised drug taken on the previous day in the first urinary morning void. Wastewater loads for BZE were of the order of 265 ± 106 mg/1000 people/day and using the correction factor of 3.59 (Castiglioni et al., 2013b), this corresponded to a cocaine consumption estimate in this smaller catchment at 950 ± 380 mg/1000 people/day. Interestingly, a high Spearman correlation (*r* = 0.90) was observed between daily lidocaine and benzoylecgonine concentrations across the week for this particular site and indicated that lidocaine occurrence may have been driven by its use as a diluent in cocaine powder (Fig. S5). Consistent, low concentrations of the opioid, oxycodone, were also observed in USA samples (average = 49 (±14) ng L^−1^), which was not present in either London or Monterrey wastewaters. It was not possible to differentiate between medicinal use and misuse of this compound using wastewater analysis. Unfortunately, more opioid standards for fentanyl, morphine, heroin, methadone and codeine were not available at the time of method development, but the speed of the MS instrument used in this method would be able cope with more MRM transitions if needed, though stability for reliable WBE back-calculations for some of these compounds is often limited.

Aside from illicit and misused drugs, several antibiotics were determined in USA wastewater samples. With respect to macrolide antibiotics, occurrence of azithromycin largely mirrored that of London, but concentrations of clarithromycin were lower on average. Lincomycin, like in London, was not detected. Trimethoprim occurrence was lower than Monterrey by three-fold on average, and roughly double that measured in London wastewater. Other notable higher occurrences of pharmaceutical residues for this site included diphenhydramine and oxazepam. Interestingly, and despite its widespread reported occurrence in the literature on a global level, carbamazepine was only detected on two days at this site and at <40 ng L^−1^. Pesticide occurrence was also measured and three were unique to this site including prometon, azoxystrobin and bupropion.

## 4 Conclusion

A rapid, direct injection LC–MS/MS method was successfully developed and validated for the quantitative determination of 135 CECs in wastewater at the ng L^−1^ concentration level. With a total analysis time of 5.0 min including re-equilibration, this enabled ∼261 injections in 24 h. With only a 10 μL injection volume, it also aided convenient and cost-effective international shipment of smaller samples and reduced the space required for archiving. Success of this method depended heavily on the use of a short, high-efficiency biphenyl LC column, the flow rate, injection volume:mobile phase ratio, MS dwell times/acquisition speed and MS detector sensitivity. The use of SPE for matrix interference removal (rather than analyte concentration) was found to be of no advantage to further enhance sensitivity. Excellent method performance was achieved over ranges of up to three orders of magnitude. When applied to influent wastewater samples from three WWTPs in London (UK), Monterrey (Mexico) and a third site in the South West USA, 56 compounds could be determined directly including pesticides, pharmaceuticals, illicit drugs and their metabolites. To our knowledge, this represents the fastest single LC–MS/MS method for direct analysis of wastewater for quantitative determinations of so many compounds at this sensitivity level. Direct analysis methods like this will likely enable rapid characterisation of CEC occurrence to monitor community-level consumption patterns and ultimately environmental risk assessment.

## CRediT authorship contribution statement

**Keng Tiong Ng:** Methodology, Formal analysis, Investigation, Writing - original draft, Writing - review & editing. **Helena Rapp-Wright:** Methodology, Validation, Investigation, Formal analysis, Writing - review & editing. **Melanie Egli:** Formal analysis, Data curation, Investigation, Validation. **Alicia Hartmann:** Formal analysis, Data curation, Investigation, Validation. **Joshua C. Steele:** Conceptualization, Methodology, Resources, Writing - review & editing. **Juan Eduardo Sosa-Hernández:** Conceptualization, Methodology, Resources, Writing - review & editing. **Elda M. Melchor-Martínez:** Writing - review & editing. **Matthew Jacobs:** Conceptualization, Resources. **Blánaid White:** Supervision, Funding acquisition. **Fiona Regan:** Conceptualization, Supervision, Funding acquisition, Writing - review & editing. **Roberto Parra-Saldivar:** Conceptualization, Resources, Supervision, Writing - review & editing. **Lewis Couchman:** Conceptualization, Writing - review & editing. **Rolf U. Halden:** Conceptualization, Resources, Supervision, Funding acquisition, Project administration, Writing - review & editing. **Leon P. Barron:** Conceptualization, Methodology, Validation, Formal analysis, Resources, Data curation, Writing - original draft, Writing - review & editing, Visualization, Supervision, Project administration, Funding acquisition.

## Declaration of Competing Interest

The authors declare that they have no known competing financial interests or personal relationships that could have appeared to influence the work reported in this paper.
