# Supplementary material for: High-throughput multi-residue quantification of contaminants of emerging concern in wastewaters enabled using direct injection liquid chromatography-tandem mass spectrometry
Source: J Hazard Mater. 2020 Nov 5;398:122933. doi: 10.1016/j.jhazmat.2020.122933 (PMC7456777; doi:10.1016/j.jhazmat.2020.122933)
Supplement: Supplementary file 1 [file mmc1.docx]

**Supplementary Information**

**HIGH-THROUGHPUT MULTI-RESIDUE QUANTIFICATION OF CONTAMINANTS OF EMERGING CONCERN IN WASTEWATERS ENABLED USING DIRECT INJECTION LIQUID CHROMATOGRAPHY-TANDEM MASS SPECTROMETRY**

Keng Tiong Ng^a,1^, Helena Rapp-Wright^a,b,1^, Melanie Egli,*^a^* Alicia Hartmann,^a,c^ Joshua C. Steele^d^, Juan Eduardo Sosa-Hernández^e^, Elda M. Melchor-Martínez^e^, Matthew Jacobs^b^, Blánaid White,^b^ Fiona Regan,^b^ Roberto Parra-Saldivar^e^, Lewis Couchman^f^, Rolf U. Halden,^d^ Leon P. Barron^a, g^*

*^a^Dept. Analytical, Environmental & Forensic Sciences, King’s College London, 150 Stamford Street, London, SE1 9NH, United Kingdom.*

*^b^DCU Water Institute and School of Chemical Sciences, Dublin City University, Glasnevin, Dublin 9, Ireland.*

*^c^Hochschule Fresenius, Limburger Straße 2, Idstein, Hessen, Germany*

*^d^Biodesign Center for Environmental Health Engineering, Biodesign Institute, Arizona State University, 1001 S McAllister Ave, Tempe, Arizona 85287-8101, United States of America.*

*^e^Tecnologico de Monterrey, Escuela de Ingenieria y Ciencias, Campus Monterrey, Ave. Eugenio Garza Sada 2501, Monterrey, Nuevo Leon 64849, Mexico.*

*^f^Analytical Services International, St George’s University of London, London, United Kingdom.*

*^g^School of Public Health, Faculty of Medicine, Imperial College London, London, United Kingdom*

*Corresponding author

Email: [leon.barron@kcl.ac.uk](mailto:leon.barron@kcl.ac.uk)

Tel: +44 20 7848 3842

Fax: +44 20 7848 4980

^1^These authors contributed equally to the work as first author.

**List of Reference Materials**

Reference materials for 2-(thiocyanomethylthio)benzothiazole, (±)4-methylethcathinone, acetamiprid, alprazolam, ametryn, amiodarone-HCl, amitriptyline, amlodipine, antipyrine, atorvastatin, atrazine, azelnidipine, (±)azithromycin, azoxystrobin, benoxacor, bensulide, (±)benzatropine, (±)benzoylecgonine (BZE), (±)betaxolol, bezafibrate, (±)bisoprolol, buspirone-HCl, carazolol, carbamazepine, carboxine, (±)carfentrazone-ethyl, carbamazepine-10,11-epoxide, celecoxib, (±)chloramphenicol, (±)cilazapril, (±)citalopram-HBr, (±)clarithromycin, (±)clodinafop-propargyl, (±)clopidogrel-HSO_4_^-^, clothianidin, clozapine, (±)cocaine, cyclouron, cycloxydim, cymoxanil, diazepam, diclofenac, diflubenzuron, (±)dimethametryn, diphenhydramine-HCl, (±)famoxadone, (±)fenoxaprop-p-ethyl, fenuron, flufenoxuron, (±)fluocinonide, (±)fluoxetine, flutamide, flutolanil, fuberidazole, hydrochlorothiazide, imidacloprid, indomethacin, isocarbamid, isradipine, (±)josamycin, (±)ketamine, (±)ketoconazole, ketotifen, (±)levamisole-HCl, (±)levocabastine-HCl, lidocaine, (±)3,4-methylenedioxymethamphetamine (MDMA), (±)meclizine-HCl, (±)medroxyprogesterone, mefenamic acid, (±)memantine-HCl, (±)mephedrone, (±)mephosfolan, (±)methamphetamine, (±)methedrone, (±)methylphenidate-HCl, (±)metoprolol, (±)nadolol, nifedipine, nordiazepam, (±)norethisterone, nortriptyline, (±)orphenadrine, oxamyl, (±)oxazepam, oxycarboxin, (±)oxycodone-HCl, picoxystrobin, (±)piperophos, pirenzepine-2HCl, pretilachlor, prodiamine, prometon, prometryn, (±)propranolol, propazine, pymetrozine, pyracarbolid, pyraclostrobin, pyraflufen-ethyl, pyridaben, risperidone, rizatriptan, ronidazole, (±)roxithromycin, (±)sertraline, simazine, (±)spinosyn A, (±)spinosyn D, (±)spiramycin, sulfadimethoxine, sulfamerazine, sulfamethazine, sulfamethoxazole, sulfamonomethoxine, sulfapyridine, sulfathiazole, sulfisoxazole, tacrine, (±)tamsulosin-HCl, (±)temazepam, terbutryn, (±)terfenadine, thiacloprid, thiamethoxam, thiazopyr, (±)timolol, (±)tramadol-HCl, (±)valsartan, (±)venlafaxine- HCl, (±)verapamil-HCl, (±)warfarin and ziprasidone-HCl were obtained from Sigma-Aldrich (Steinheim, Germany). Trimethoprim was acquired from Fluka (Buchs, Switzerland).

Stable isotope-labelled standards (SIL-IS) including amitriptyline-d_3_-HCl, (±)BZE-d_3_, clothianidin-d_3_, (±)cocaine-d_3_, diazepam-d_6_, (±)fluoxetine-d_6_, (±)ketamine-d_4_-HCl, (±)MDMA-d_5_, (±)lorazepam-d_4_, (±)methylphenidate-d_9_, nordiazepam-d_5_, nortriptyline-d_3_-HCl, (±)oxazepam-d_5_, risperidone-d_4_, (±)sertraline-d_3_-HCl, (±)temazepam-d_5_, thiamethoxam-d_3_, (±)tramadol-^13^C,d_3_-HCl, (±)venlafaxine-d_6_-HCl, were purchased from Sigma Aldrich. (±)Betaxolol-d_7_-HCl, celecoxib-d_7_, (±)clarithromycin-d_3_, lidocaine-d_10_-HCl, (±)metoprolol-d_7_-HCl, sulfamethazine-d_4_, trimethoprim-d_3_ and (±)verapamil-d_3_-HCl were ordered from QMX (Essex, UK).

**Table S1.** MRM scheduling and other parameters for the optimised direct injection LC-MS/MS method

| Compounds | Precursor (m/z) | Transition (m/z) | Polarity | Pause Time (ms) | Dwell Time (ms) | Q1 Pre Bias | Collision Energy (V) | Q3 Pre Bias | Retention Time Window Range (min) | |
| --- | --- | --- | --- | --- | --- | --- | --- | --- | --- | --- |
|  |  |  |  |  |  | (V) |  | (V) |  |  |
| 2-(Thiocyanomethylthio)  benzothiazole | 238.7 | 180.1 | + | 2 | 2 | -27 | -14 | -19 | 2.44 | 3.04 |
|  |  | 136.1 | + | 2 | 2 | -29 | -26 | -14 |  |  |
| 4-Methylethcathinone | 192.1 | 174.2 | + | 2 | 5 | -23 | -16 | -18 | 0.50 | 1.10 |
|  |  | 144.1 | + | 2 | 5 | -10 | -29 | -25 |  |  |
| Acetamiprid | 233 | 126.1 | + | 2 | 13 | -16 | -21 | -13 | 1.22 | 1.82 |
| Alprazolam | 309.1 | 281.1 | + | 2 | 2 | -16 | -26 | -22 | 2.14 | 2.74 |
|  |  | 205.1 | + | 2 | 2 | -16 | -40 | -23 |  |  |
| Ametryn | 228.1 | 186.1 | + | 2 | 4 | -11 | -20 | -20 | 1.27 | 1.87 |
|  |  | 96.1 | + | 2 | 4 | -23 | -28 | -19 |  |  |
| Amiodarone | 645.8 | 58.2 | + | 2 | 20 | -32 | -49 | -27 | 3.07 | 3.67 |
|  |  | 100.2 | + | 2 | 20 | -32 | -30 | -10 |  |  |
| Amitriptyline | 278.2 | 91.1 | + | 2 | 4 | -13 | -27 | -19 | 1.97 | 2.57 |
|  |  | 105.1 | + | 2 | 4 | -13 | -25 | -21 |  |  |
| Amitriptyline-d3 | 281.1 | 233.2 | + | 2 | 4 | -19 | -17 | -25 | 1.96 | 2.56 |
|  |  | 105.2 | + | 2 | 4 | -10 | -23 | -19 |  |  |
| Amlodipine | 409.1 | 238.2 | + | 2 | 6 | -15 | -12 | -24 | 1.94 | 2.54 |
| Antipyrine | 188.9 | 77.2 | + | 2 | 5 | -13 | -40 | -30 | 0.83 | 1.43 |
|  |  | 56.2 | + | 2 | 5 | -13 | -32 | -23 |  |  |
| Atorvastatin | 559.1 | 440.3 | + | 2 | 5 | -20 | -23 | -16 | 2.88 | 3.48 |
|  |  | 250.2 | + | 2 | 5 | -20 | -43 | -27 |  |  |
| Atrazine | 216.1 | 174.1 | + | 2 | 14 | -11 | -18 | -18 | 1.48 | 2.08 |
| Azelnidipine | 583.2 | 167.2 | + | 2 | 2 | -40 | -27 | -30 | 2.80 | 3.40 |
|  |  | 165.2 | + | 2 | 2 | -40 | -55 | -16 |  |  |
| Azithromycin | 749.6 | 591.4 | + | 2 | 2 | -28 | -30 | -22 | 1.30 | 1.90 |
|  |  | 158.3 | + | 2 | 2 | -28 | -40 | -16 |  |  |
| Azoxystrobin | 404.2 | 372.1 | + | 2 | 2 | -21 | -16 | -27 | 2.73 | 3.33 |
|  |  | 344.1 | + | 2 | 2 | -21 | -26 | -24 |  |  |
| Benoxacor | 260 | 149.2 | + | 2 | 2 | -30 | -18 | -27 | 2.20 | 2.80 |
|  |  | 134.1 | + | 2 | 2 | -30 | -29 | -25 |  |  |
| Bensulide | 398.1 | 158.1 | + | 2 | 1 | -20 | -24 | -16 | 3.00 | 3.60 |
|  |  | 217.9 | + | 2 | 1 | -20 | -17 | -23 |  |  |
| Benzatropine | 308.1 | 167.2 | + | 2 | 5 | -11 | -30 | -17 | 2.01 | 2.61 |
|  |  | 265.2 | + | 2 | 5 | -21 | -52 | -17 |  |  |
| Benzoylecgonine, BZE | 290.1 | 168.2 | + | 2 | 11 | -20 | -19 | -18 | 0.77 | 1.37 |
| Benzoylecgonine, BZE-d3 | 293.1 | 171.2 | + | 2 | 11 | -30 | -20 | -18 | 0.77 | 1.37 |
| Betaxolol | 308.3 | 116.2 | + | 2 | 4 | -15 | -21 | -21 | 1.38 | 1.98 |
|  |  | 72.2 | + | 2 | 4 | -21 | -24 | -27 |  |  |
| Betaxolol-d7 | 315.2 | 123.3 | + | 2 | 4 | -23 | -22 | -22 | 1.37 | 1.97 |
|  |  | 105.3 | + | 2 | 4 | -23 | -24 | -11 |  |  |
| Bezafibrate | 360.2 | 274 | - | 2 | 5 | 18 | 17 | 12 | 2.27 | 2.87 |
|  |  | 154.1 | - | 2 | 5 | 20 | 29 | 15 |  |  |
| Bisoprolol | 326.2 | 116.2 | + | 2 | 5 | -23 | -19 | -21 | 1.15 | 1.75 |
|  |  | 74.2 | + | 2 | 5 | -12 | -26 | -29 |  |  |
| Bupropion | 240.1 | 184.2 | + | 2 | 4 | -29 | -13 | -12 | 0.98 | 1.58 |
|  |  | 131.2 | + | 2 | 4 | -27 | -26 | -13 |  |  |
| Buspirone | 386.1 | 122.2 | + | 2 | 2 | -26 | -30 | -23 | 1.49 | 2.09 |
|  |  | 109.2 | + | 2 | 2 | -25 | -46 | -11 |  |  |
| Carazolol | 299.1 | 116.2 | + | 2 | 6 | -21 | -21 | -20 | 1.32 | 1.92 |
|  |  | 222.2 | + | 2 | 6 | -21 | -21 | -24 |  |  |
| Carbamazepine | 237.1 | 194 | + | 2 | 4 | -12 | -20 | -23 | 1.71 | 2.31 |
|  |  | 192.1 | + | 2 | 4 | -12 | -25 | -22 |  |  |
| Carboxine | 236 | 143.1 | + | 2 | 13 | -26 | -15 | -14 | 1.77 | 2.37 |
| Carfentrazone-ethyl | 412.1 | 346 | + | 2 | 2 | -19 | -17 | -24 | 2.81 | 3.41 |
|  |  | 366 | + | 2 | 2 | -19 | -19 | -21 |  |  |
| Carbamazepine epoxide | 252.9 | 180.2 | + | 2 | 4 | -17 | -29 | -18 | 1.40 | 2.00 |
|  |  | 236.2 | + | 2 | 4 | -17 | -12 | -16 |  |  |
| Celecoxib | 382.1 | 362.1 | + | 2 | 4 | -11 | -28 | -25 | 2.73 | 3.33 |
|  |  | 300.2 | + | 2 | 4 | -18 | -28 | -21 |  |  |
| Celecoxib-d7 | 389 | 369.1 | + | 2 | 2 | -19 | -29 | -25 | 2.72 | 3.32 |
|  |  | 289.2 | + | 2 | 2 | -26 | -37 | -30 |  |  |
| Chloramphenicol | 322.7 | 152.2 | - | 2 | 5 | 16 | 17 | 15 | 1.08 | 1.68 |
|  |  | 257 | - | 2 | 5 | 17 | 11 | 25 |  |  |
| Cilazapril | 418 | 211.2 | + | 2 | 5 | -20 | -20 | -20 | 1.83 | 2.43 |
|  |  | 70.2 | + | 2 | 5 | -15 | -46 | -12 |  |  |
| Citalopram | 325.1 | 109.2 | + | 2 | 4 | -22 | -26 | -20 | 1.63 | 2.23 |
|  |  | 262.2 | + | 2 | 4 | -24 | -20 | -30 |  |  |
| Clarithromycin | 748.2 | 158.2 | + | 2 | 2 | -36 | -34 | -10 | 1.95 | 2.55 |
|  |  | 290.4 | + | 2 | 2 | -36 | -20 | -22 |  |  |
| Clarithromycin-d3 | 751.2 | 161.2 | + | 2 | 2 | -38 | -28 | -16 | 1.95 | 2.55 |
|  |  | 593.4 | + | 2 | 2 | -38 | -22 | -22 |  |  |
| Clozapine | 327.2 | 270.2 | + | 2 | 2 | -16 | -23 | -20 | 1.18 | 1.78 |
|  |  | 192.1 | + | 2 | 2 | -16 | -45 | -14 |  |  |
| Cocaine | 304.2 | 182.3 | + | 2 | 5 | -11 | -21 | -14 | 1.09 | 1.69 |
|  |  | 82.1 | + | 2 | 5 | -11 | -35 | -17 |  |  |
| Cocaine-d3 | 307.1 | 185.2 | + | 2 | 5 | -22 | -21 | -19 | 1.09 | 1.69 |
|  |  | 77.2 | + | 2 | 5 | -15 | -54 | -14 |  |  |
| Cyclouron | 199.1 | 72.2 | + | 2 | 3 | -14 | -25 | -13 | 1.64 | 2.24 |
|  |  | 89.2 | + | 2 | 3 | -22 | -14 | -16 |  |  |
| Cycloxyidim | 326.1 | 280.2 | + | 2 | 5 | -16 | -14 | -19 | 2.97 | 3.57 |
|  |  | 180.2 | + | 2 | 5 | -12 | -22 | -19 |  |  |
| Cymoxanil | 199.2 | 111.2 | + | 2 | 3 | -23 | -19 | -18 | 0.93 | 1.53 |
|  |  | 83 | + | 2 | 3 | -23 | -27 | -17 |  |  |
| Diazepam | 285.1 | 154.1 | + | 2 | 2 | -11 | -28 | -18 | 2.29 | 2.89 |
|  |  | 193.1 | + | 2 | 2 | -11 | -29 | -23 |  |  |
| Diazepam-d6 | 289.9 | 198.2 | + | 2 | 2 | -19 | -33 | -21 | 2.27 | 2.87 |
|  |  | 154.2 | + | 2 | 2 | -19 | -27 | -15 |  |  |
| Diclofenac | 296 | 215.1 | + | 2 | 6 | -15 | -20 | -16 | 2.68 | 3.28 |
|  |  | 214 | + | 2 | 6 | -15 | -40 | -24 |  |  |
| Diflubenzuron | 311 | 158.1 | + | 2 | 1 | -16 | -16 | -29 | 2.60 | 3.20 |
|  |  | 141.1 | + | 2 | 1 | -16 | -32 | -24 |  |  |
| Dimethametryn | 256.1 | 186.2 | + | 2 | 2 | -28 | -22 | -30 | 1.79 | 2.39 |
|  |  | 68.1 | + | 2 | 2 | -13 | -44 | -11 |  |  |
| Diphenhydramine | 256 | 167.1 | + | 2 | 2 | -30 | -11 | -12 | 1.52 | 2.12 |
|  |  | 152 | + | 2 | 2 | -30 | -40 | -17 |  |  |
| Famoxadone | 375.8 | 196.1 | + | 2 | 6 | -25 | -21 | -20 | 3.07 | 3.67 |
| Fenoxaprop-ethyl | 362.1 | 288.1 | + | 2 | 2 | -18 | -18 | -21 | 3.10 | 3.70 |
|  |  | 121.1 | + | 2 | 2 | -19 | -33 | -13 |  |  |
| Fenuron | 165 | 72.2 | + | 2 | 5 | -18 | -22 | -28 | 0.50 | 1.10 |
|  |  | 46.1 | + | 2 | 5 | -18 | -14 | -18 |  |  |
| Flufenoxuron | 489.1 | 158.1 | + | 2 | 2 | -15 | -21 | -17 | 3.14 | 3.74 |
|  |  | 141 | + | 2 | 2 | -24 | -39 | -15 |  |  |
| Fluocinonide | 494.9 | 337.2 | + | 2 | 6 | -23 | -19 | -25 | 2.69 | 3.29 |
| Fluoxetine | 310.2 | 44.2 | + | 2 | 2 | -15 | -16 | -17 | 1.89 | 2.49 |
|  |  | 148.2 | + | 2 | 2 | -15 | -10 | -11 |  |  |
| Fluoxetine-d6 | 316.1 | 44.2 | + | 2 | 2 | -20 | -15 | -20 | 1.88 | 2.48 |
|  |  | 154.3 | + | 2 | 2 | -15 | -9 | -16 |  |  |
| Flutamide | 275 | 202 | - | 2 | 2 | 14 | 23 | 20 | 2.20 | 2.80 |
|  |  | 205 | - | 2 | 2 | 13 | 21 | 23 |  |  |
| Flutolanil | 324.3 | 242.2 | + | 2 | 2 | -15 | -26 | -16 | 2.56 | 3.16 |
|  |  | 262.2 | + | 2 | 2 | -15 | -19 | -18 |  |  |
| Fuberidazole | 184.9 | 157.2 | + | 2 | 5 | -23 | -21 | -16 | 0.17 | 0.77 |
|  |  | 156.2 | + | 2 | 5 | -23 | -27 | -16 |  |  |
| Hydrochlorothiazide | 296 | 269 | - | 2 | 10 | 11 | 19 | 11 | 0.14 | 0.74 |
| Imidacloprid | 256.1 | 175.1 | + | 2 | 5 | -13 | -19 | -20 | 1.06 | 1.66 |
|  |  | 209.1 | + | 2 | 5 | -13 | -18 | -10 |  |  |
| Indomethacin | 356.5 | 312.3 | - | 2 | 14 | 18 | 11 | 10 | 2.81 | 3.41 |
| Isocarbamid | 186.1 | 87 | + | 2 | 2 | -20 | -16 | -15 | 0.63 | 1.23 |
|  |  | 44.2 | + | 2 | 2 | -20 | -32 | -17 |  |  |
| Isradipine | 370.2 | 119 | - | 2 | 2 | 14 | 16 | 11 | 2.52 | 3.12 |
|  |  | 250 | - | 2 | 2 | 11 | 16 | 17 |  |  |
| Josamycin | 828.5 | 109.3 | + | 2 | 6 | -30 | -47 | -27 | 2.34 | 2.94 |
| Ketamine | 238.1 | 125 | + | 2 | 5 | -12 | -26 | -26 | 0.75 | 1.35 |
|  |  | 207 | + | 2 | 5 | -12 | -15 | -15 |  |  |
| Ketamine-d4 | 242.1 | 129.2 | + | 2 | 5 | -25 | -28 | -26 | 0.73 | 1.33 |
|  |  | 211.3 | + | 2 | 5 | -28 | -15 | -10 |  |  |
| Ketoconazole | 533 | 491.1 | + | 2 | 4 | -36 | -31 | -24 | 2.18 | 2.78 |
|  |  | 82.2 | + | 2 | 4 | -36 | -47 | -14 |  |  |
| Ketotifen | 310 | 96.2 | + | 2 | 10 | -22 | -24 | -18 | 1.40 | 2.00 |
| Levamisole | 207.1 | 180 | + | 2 | 10 | -10 | -24 | -20 | 0.24 | 0.84 |
|  |  | 91.1 | + | 2 | 10 | -10 | -41 | -17 |  |  |
| Levocabastine | 421.4 | 174.2 | + | 2 | 6 | -20 | -32 | -18 | 1.90 | 2.50 |
|  |  | 70.2 | + | 2 | 6 | -15 | -37 | -12 |  |  |
| Lidocaine | 235 | 86.1 | + | 2 | 5 | -26 | -19 | -18 | 0.21 | 0.81 |
|  |  | 58.1 | + | 2 | 5 | -26 | -45 | -12 |  |  |
| Lidocaine-d10 | 245.2 | 96.3 | + | 2 | 13 | -29 | -22 | -17 | 0.21 | 0.81 |
| MDMA | 194.1 | 163.1 | + | 2 | 5 | -10 | -13 | -12 | 0.25 | 0.85 |
|  |  | 105.1 | + | 2 | 5 | -10 | -25 | -22 |  |  |
| MDMA-d5 | 199.1 | 165.3 | + | 2 | 5 | -22 | -14 | -29 | 0.24 | 0.84 |
|  |  | 107.2 | + | 2 | 5 | -10 | -25 | -22 |  |  |
| Meclizine | 391.1 | 201.1 | + | 2 | 1 | -28 | -20 | -22 | 2.71 | 3.31 |
|  |  | 165.1 | + | 2 | 1 | -19 | -55 | -17 |  |  |
| Medroxyprogesterone | 345.1 | 123.2 | + | 2 | 2 | -23 | -26 | -21 | 2.71 | 3.31 |
|  |  | 97.2 | + | 2 | 2 | -23 | -27 | -10 |  |  |
| Mefenamic acid | 240 | 196.2 | - | 2 | 12 | 17 | 18 | 12 | 2.82 | 3.42 |
| Memantine | 180.3 | 163.3 | + | 2 | 6 | -19 | -18 | -17 | 0.95 | 1.55 |
|  |  | 107.3 | + | 2 | 6 | -20 | -26 | -20 |  |  |
| Mephedrone | 178.3 | 145.1 | + | 1 | 10 | -20 | -21 | -29 | 0.31 | 0.91 |
|  |  | 160.3 | + | 1 | 10 | -20 | -16 | -11 |  |  |
| Mephosfolan | 270.1 | 140 | + | 2 | 12 | -13 | -25 | -14 | 1.50 | 2.10 |
| Methamphetamine | 150.1 | 91.1 | + | 2 | 5 | -10 | -19 | -18 | 0.00 | 0.59 |
|  |  | 119.1 | + | 2 | 5 | -10 | -10 | -24 |  |  |
| Methedrone | 194.3 | 176.2 | + | 2 | 5 | -20 | -15 | -29 | 0.22 | 0.82 |
|  |  | 161.1 | + | 2 | 5 | -21 | -21 | -16 |  |  |
| Methylphenidate | 234.2 | 84.1 | + | 2 | 6 | -11 | -20 | -17 | 0.87 | 1.47 |
|  |  | 56.1 | + | 2 | 6 | -11 | -45 | -22 |  |  |
| Methylphenidate-d9 | 243.3 | 93.3 | + | 5 | 6 | -28 | -24 | -17 | 0.85 | 1.45 |
|  |  | 61.2 | + | 2 | 6 | -27 | -50 | -23 |  |  |
| Metoprolol | 268.2 | 116.2 | + | 2 | 5 | -30 | -21 | -21 | 0.80 | 1.40 |
|  |  | 159.1 | + | 2 | 5 | -10 | -22 | -16 |  |  |
| Metoprolol-d7 | 275.2 | 123.2 | + | 2 | 5 | -22 | -21 | -23 | 0.79 | 1.39 |
|  |  | 105.2 | + | 2 | 5 | -29 | -22 | -19 |  |  |
| Nadolol | 310.3 | 254.2 | + | 2 | 3 | -21 | -17 | -17 | 0.34 | 0.94 |
|  |  | 236.2 | + | 2 | 3 | -21 | -21 | -25 |  |  |
| Nifedipine | 345.2 | 222.1 | - | 2 | 3 | 10 | 10 | 15 | 2.22 | 2.82 |
|  |  | 122 | - | 2 | 3 | 13 | 12 | 27 |  |  |
| Nifedipine-d4 | 349 | 222.1 | - | 2 | 3 | 10 | 10 | 24 | 2.22 | 2.82 |
|  |  | 126.1 | - | 2 | 3 | 13 | 12 | 28 |  |  |
| Nordiazepam | 270.8 | 140.2 | + | 2 | 2 | -18 | -28 | -14 | 1.86 | 2.46 |
|  |  | 208.1 | + | 2 | 2 | -10 | -28 | -14 |  |  |
| Nordiazepam-d5 | 275.9 | 213.3 | + | 2 | 2 | -10 | -28 | -22 | 1.84 | 2.44 |
|  |  | 140.2 | + | 2 | 2 | -10 | -31 | -14 |  |  |
| Norethisterone | 299.1 | 109.2 | + | 2 | 2 | -21 | -26 | -19 | 2.56 | 3.16 |
|  |  | 231.2 | + | 2 | 2 | -11 | -20 | -11 |  |  |
| Nortriptyline | 264.2 | 233.1 | + | 2 | 4 | -10 | -15 | -17 | 1.92 | 2.52 |
|  |  | 91.1 | + | 2 | 4 | -10 | -25 | -18 |  |  |
| Nortriptyline-d3 | 267 | 233.2 | + | 2 | 4 | -18 | -15 | -16 | 1.92 | 2.52 |
|  |  | 105.2 | + | 2 | 4 | -18 | -22 | -19 |  |  |
| Orphenadrine | 270.1 | 181.2 | + | 2 | 4 | -29 | -13 | -29 | 1.71 | 2.31 |
|  |  | 166.1 | + | 2 | 4 | -10 | -28 | -30 |  |  |
| Oxamyl | 237.1 | 72.1 | + | 2 | 4 | -12 | -12 | -13 | 0.24 | 0.84 |
|  |  | 90.2 | + | 2 | 4 | -12 | -10 | -17 |  |  |
| Oxazepam | 287.2 | 241.2 | + | 2 | 5 | -11 | -24 | -19 | 1.89 | 2.49 |
|  |  | 269.1 | + | 2 | 5 | -11 | -15 | -22 |  |  |
| Oxazepam-d5 | 292 | 246.2 | + | 2 | 5 | -22 | -24 | -26 | 1.87 | 2.47 |
|  |  | 274.1 | + | 2 | 5 | -11 | -17 | -13 |  |  |
| Oxycarboxin | 268.1 | 175 | + | 2 | 5 | -13 | -14 | -18 | 1.20 | 1.80 |
|  |  | 147 | + | 2 | 5 | -13 | -27 | -30 |  |  |
| Oxycodone | 316.2 | 298.2 | + | 2 | 20 | -16 | -19 | -22 | 0.40 | 1.00 |
|  |  | 241.1 | + | 2 | 20 | -16 | -30 | -18 |  |  |
| Picoxystrobin | 368 | 145.2 | + | 2 | 1 | -25 | -22 | -15 | 2.92 | 3.52 |
|  |  | 205.2 | + | 2 | 1 | -25 | -10 | -14 |  |  |
| Piperophos | 353.9 | 171.1 | + | 2 | 2 | -23 | -22 | -18 | 3.07 | 3.67 |
|  |  | 255.1 | + | 2 | 2 | -22 | -14 | -12 |  |  |
| Pirenzipine | 352.1 | 113.3 | + | 2 | 5 | -26 | -22 | -22 | 0.50 | 1.10 |
|  |  | 70.2 | + | 2 | 5 | -10 | -46 | -12 |  |  |
| Pretilachlor | 312.2 | 252.1 | + | 2 | 2 | -16 | -17 | -17 | 2.97 | 3.57 |
|  |  | 176.1 | + | 2 | 2 | -16 | -29 | -18 |  |  |
| Prodiamine | 349.2 | 232 | - | 2 | 2 | 10 | 24 | 10 | 3.10 | 3.70 |
|  |  | 216 | - | 2 | 2 | 13 | 29 | 14 |  |  |
| Prometon | 226.1 | 184.2 | + | 2 | 5 | -11 | -20 | -12 | 1.08 | 1.68 |
|  |  | 142.2 | + | 2 | 5 | -11 | -23 | -14 |  |  |
| Prometryn | 242.2 | 158.1 | + | 2 | 2 | -12 | -25 | -16 | 1.52 | 2.12 |
|  |  | 200 | + | 2 | 2 | -12 | -20 | -22 |  |  |
| Propanolol | 260.1 | 116.2 | + | 2 | 4 | -30 | -19 | -21 | 1.39 | 1.99 |
|  |  | 183.2 | + | 2 | 4 | -10 | -20 | -18 |  |  |
| Propazine | 230.1 | 188.2 | + | 2 | 4 | -26 | -19 | -20 | 1.80 | 2.40 |
|  |  | 146.1 | + | 2 | 4 | -25 | -24 | -25 |  |  |
| Pymetrozine | 218.1 | 105.1 | + | 2 | 6 | -11 | -21 | -21 | 0.00 | 0.45 |
|  |  | 79 | + | 2 | 6 | -11 | -45 | -15 |  |  |
| Pyracarbolid | 218.1 | 125.1 | + | 2 | 3 | -24 | -18 | -24 | 1.56 | 2.16 |
|  |  | 97.1 | + | 2 | 3 | -24 | -28 | -17 |  |  |
| Pyraclostrobin | 390.1 | 194.1 | + | 2 | 2 | -19 | -15 | -21 | 3.09 | 3.69 |
|  |  | 163.1 | + | 2 | 2 | -19 | -27 | -17 |  |  |
| Pyraflufen-ethyl | 413 | 339 | + | 2 | 2 | -21 | -20 | -23 | 2.97 | 3.57 |
|  |  | 253.1 | + | 2 | 2 | -21 | -35 | -26 |  |  |
| Pyridaben | 364.8 | 147.2 | + | 2 | 3 | -17 | -25 | -15 | 3.16 | 3.76 |
|  |  | 309.1 | + | 2 | 3 | -18 | -14 | -15 |  |  |
| Risperidone | 411.2 | 191.1 | + | 2 | 10 | -12 | -31 | -23 | 1.37 | 1.97 |
|  |  | 69.1 | + | 2 | 10 | -12 | -50 | -27 |  |  |
| Risperidone-d4 | 415.2 | 195.2 | + | 2 | 10 | -29 | -30 | -21 | 1.36 | 1.96 |
| Rizatriptan | 270.1 | 201.2 | + | 2 | 5 | -10 | -14 | -21 | 0.21 | 0.81 |
|  |  | 158.2 | + | 2 | 5 | -20 | -21 | -16 |  |  |
| Ronidazole | 201 | 140.2 | + | 2 | 11 | -14 | -13 | -14 | 0.14 | 0.74 |
| Roxithromycin | 837.3 | 679.4 | + | 2 | 4 | -24 | -22 | -24 | 2.10 | 2.70 |
|  |  | 158.1 | + | 2 | 4 | -24 | -34 | -16 |  |  |
| Sertraline | 306.1 | 159 | + | 2 | 4 | -15 | -26 | -18 | 2.13 | 2.73 |
|  |  | 275.1 | + | 2 | 4 | -15 | -15 | -20 |  |  |
| Sertraline-d3 | 309 | 159.1 | + | 2 | 4 | -30 | -28 | -30 | 2.13 | 2.73 |
|  |  | 275.1 | + | 2 | 4 | -11 | -14 | -29 |  |  |
| Simazine | 202.1 | 104 | + | 2 | 5 | -23 | -25 | -19 | 1.14 | 1.74 |
|  |  | 68.1 | + | 2 | 5 | -23 | -32 | -27 |  |  |
| Spinosyn A | 732.6 | 142.1 | + | 2 | 5 | -22 | -34 | -29 | 2.79 | 3.39 |
|  |  | 98 | + | 2 | 5 | -22 | -40 | -19 |  |  |
| Spinosyn D | 746.6 | 142.1 | + | 2 | 6 | -22 | -36 | -15 | 2.93 | 3.53 |
|  |  | 98 | + | 2 | 6 | -22 | -40 | -19 |  |  |
| Spiramycin | 843.5 | 174.2 | + | 2 | 10 | -24 | -36 | -11 | 1.41 | 2.01 |
| Sulfadimethoxine | 311.1 | 156 | + | 1 | 10 | -16 | -20 | -17 | 1.43 | 2.03 |
|  |  | 92.1 | + | 1 | 10 | -16 | -32 | -17 |  |  |
| Sulfamerazine | 265.1 | 92.1 | + | 2 | 3 | -13 | -34 | -17 | 1.92 | 2.52 |
|  |  | 156 | + | 2 | 3 | -13 | -17 | -16 |  |  |
| Sulfamethazine | 278.9 | 186.1 | + | 2 | 6 | -18 | -17 | -19 | 0.81 | 1.41 |
|  |  | 124.2 | + | 2 | 6 | -10 | -24 | -12 |  |  |
| Sulfamethazine-d4 | 282.8 | 186.2 | + | 2 | 3 | -13 | -20 | -19 | 0.80 | 1.40 |
|  |  | 124.2 | + | 2 | 3 | -17 | -25 | -27 |  |  |
| Sulfamethoxazole | 254.1 | 156 | + | 2 | 6 | -12 | -18 | -16 | 1.04 | 1.64 |
|  |  | 92.2 | + | 2 | 6 | -12 | -31 | -16 |  |  |
| Sulfamonomethoxine | 281.1 | 156.1 | + | 2 | 4 | -14 | -18 | -17 | 0.94 | 1.54 |
|  |  | 92.2 | + | 2 | 4 | -14 | -33 | -18 |  |  |
| Sulfapyridine | 250 | 156 | + | 2 | 4 | -23 | -17 | -16 | 0.39 | 0.99 |
|  |  | 92.1 | + | 2 | 4 | -22 | -32 | -18 |  |  |
| Sulfathiazole | 256 | 156 | + | 2 | 8 | -24 | -16 | -16 | 0.42 | 1.02 |
|  |  | 92.2 | + | 2 | 8 | -24 | -27 | -17 |  |  |
| Sulfisoxazole | 268 | 156.1 | + | 2 | 13 | -30 | -15 | -16 | 1.14 | 1.74 |
|  |  | 113.2 | + | 2 | 13 | -10 | -16 | -11 |  |  |
| Tacrine | 199 | 171.2 | + | 2 | 6 | -23 | -30 | -17 | 0.73 | 1.33 |
|  |  | 144.1 | + | 2 | 6 | -14 | -36 | -28 |  |  |
| Tamsulosin | 409.1 | 228.1 | + | 2 | 4 | -28 | -24 | -24 | 1.43 | 2.03 |
|  |  | 271.2 | + | 2 | 4 | -28 | -20 | -13 |  |  |
| Temazepam | 301.1 | 255.1 | + | 2 | 4 | -11 | -25 | -30 | 2.17 | 2.77 |
|  |  | 283.2 | + | 2 | 4 | -11 | -13 | -23 |  |  |
| Temazepam-d5 | 306 | 260.1 | + | 2 | 10 | -11 | -24 | -17 | 2.16 | 2.76 |
| Terbutryn | 242.1 | 186.1 | + | 2 | 2 | -28 | -20 | -20 | 1.58 | 2.18 |
|  |  | 158.2 | + | 2 | 2 | -29 | -24 | -27 |  |  |
| Terfenadine | 472.4 | 436.3 | + | 2 | 6 | -24 | -28 | -22 | 2.69 | 3.29 |
|  |  | 454.3 | + | 2 | 6 | -13 | -22 | -22 |  |  |
| Thiacloprid | 253.1 | 126.1 | + | 2 | 4 | -13 | -22 | -25 | 1.53 | 2.13 |
|  |  | 90.1 | + | 2 | 4 | -13 | -38 | -18 |  |  |
| Thiamethoxam | 292 | 211.1 | + | 2 | 2 | -14 | -13 | -23 | 0.71 | 1.31 |
|  |  | 181 | + | 2 | 2 | -14 | -24 | -19 |  |  |
| Thiamethoxam-d3 | 296.6 | 214.1 | + | 2 | 3 | -11 | -12 | -23 | 0.68 | 1.28 |
|  |  | 184.1 | + | 2 | 3 | -14 | -24 | -19 |  |  |
| Thiazopyr | 397 | 377.1 | + | 2 | 6 | -14 | -23 | -18 | 2.86 | 3.46 |
| Timolol | 317.1 | 261.1 | + | 2 | 13 | -16 | -17 | -30 | 0.77 | 1.37 |
| Tramadol | 264.1 | 58.2 | + | 2 | 14 | -19 | -16 | -22 | 0.82 | 1.42 |
| Tramadol-13C1, d3 | 268.3 | 58.2 | + | 2 | 14 | -13 | -23 | -24 | 0.81 | 1.41 |
| Trimethoprim | 291.1 | 230.1 | + | 2 | 5 | -30 | -25 | -26 | 0.45 | 1.05 |
|  |  | 123.2 | + | 2 | 5 | -15 | -28 | -25 |  |  |
| Trimethoprim-d3 | 294.1 | 230.2 | + | 2 | 5 | -20 | -25 | -25 | 0.44 | 1.04 |
|  |  | 123.2 | + | 2 | 5 | -11 | -26 | -12 |  |  |
| Valsartan | 436.4 | 291.2 | + | 2 | 5 | -15 | -18 | -14 | 2.50 | 3.10 |
|  |  | 235.2 | + | 2 | 5 | -12 | -17 | -16 |  |  |
| Venlafaxine | 278.2 | 58.1 | + | 2 | 5 | -13 | -19 | -22 | 1.13 | 1.73 |
|  |  | 260.2 | + | 2 | 5 | -13 | -15 | -20 |  |  |
| Venlafaxine-d6 | 284.2 | 64.2 | + | 2 | 5 | -20 | -23 | -26 | 1.13 | 1.73 |
|  |  | 260.2 | + | 2 | 5 | -13 | -15 | -20 |  |  |
| Verapamil | 455.2 | 165.2 | + | 2 | 2 | -30 | -29 | -30 | 1.99 | 2.59 |
|  |  | 414.4 | + | 2 | 2 | -16 | -16 | -15 |  |  |
| Verapamil-d3 | 458.2 | 165.2 | + | 2 | 2 | -30 | -29 | -29 | 1.99 | 2.59 |
|  |  | 306.3 | + | 2 | 2 | -30 | -26 | -15 |  |  |
| Warfarin | 309.2 | 163.1 | + | 2 | 5 | -15 | -16 | -19 | 2.36 | 2.96 |
|  |  | 251.1 | + | 2 | 5 | -15 | -20 | -29 |  |  |
| Ziprasidone | 413.1 | 194.1 | + | 2 | 2 | -20 | -30 | -20 | 1.75 | 2.35 |

**Table S2.** ESI conditions and dwell/loop time summary

| **ESI Interface Conditions** | |
| --- | --- |
| Nerbulising gas flow, L/min | 3 |
| Heating gas flow, L/min | 10 |
| Interface temperature, °C | 300 |
| DL temperature, °C | 250 |
| Heat block temperature, °C | 400 |
| Drying gas flow, L/min | 10 |
|  |  |
|  |  |
| **Dwell Time/Loop Time** | |
| Maximum event | 49 |
| Maximum dwell time, ms | 20 |
| Minimum dwell time, ms | 1 |
| Maximum loop time, s | 0.572 |

**Table S3.** Percentage ±standard deviation (SD) of analyte peak area measured after removal of sample matrix by SPE relative to area measured with direct injection (no SPE) in artificial freshwater and influent wastewater matrix for a selection of 105 compounds. SPE cartridge key: **Alu** - Phenomenex Strata^TM^ Alumina-N; **CN-E** - Agilent Bond Elut CN-E; **WAX** - Thermo Sola^TM^ WAX; **AX** - Thermo Hypersep^TM^ Retain AX; **SCX** - Thermo Hypersep^TM^ SCX; **CX** - Thermo Hypersep^TM^ Retain CX; **PEP** - Thermo Hypersep^TM^ Retain PEP. nd – not detected

| Compound | Artificial Freshwater (n=3) | | | | | | | | | | | | | |  | Untreated Wastewater (n=3) | | | | | | | | | | | | | |
| --- | --- | --- | --- | --- | --- | --- | --- | --- | --- | --- | --- | --- | --- | --- | --- | --- | --- | --- | --- | --- | --- | --- | --- | --- | --- | --- | --- | --- | --- |
|  | Alu. | | CN-E | | WAX | | AX | | SCX | | CX | | PEP | |  | Alu. | | CN-E | | WAX | | AX | | SCX | | CX | | PEP | |
|  | % | SD | % | SD | % | SD | % | SD | % | SD | % | SD | % | SD |  | % | SD | % | SD | % | SD | % | SD | % | SD | % | SD | % | SD |
| 2-(Thiocyanomethylthio)  benzothiazole | 6 | 3 | 5 | 2 | 5 | 1 | 5 | 1 | 5 | 1 | 5 | 2 | 5 | 2 |  | 139 | 38 | 116 | 33 | 107 | 28 | 120 | 25 | 163 | 43 | 144 | 55 | 156 | 43 |
| 4-MEC | 25 | 4 | 0 | 0 | 28 | 5 | 7 | 1 | 1 | 0 | 0 | 0 | 0 | 0 |  | 60 | 7 | 1 | 0 | 11 | 6 | 4 | 1 | 2 | 1 | 1 | 0 | 1 | 0 |
| Acetamiprid | 27 | 4 | 0 | 0 | 0 | 0 | 0 | 0 | 0 | 0 | 0 | 0 | 0 | 0 |  | 26 | 3 | 0 | 0 | 0 | 0 | 0 | 0 | 0 | 0 | 0 | 0 | 0 | 0 |
| Ametryn | 8 | 3 | 0 | 0 | 0 | 0 | 0 | 0 | 0 | 0 | 0 | 0 | 0 | 0 |  | 41 | 9 | 0 | 0 | 0 | 0 | 1 | 0 | 1 | 0 | 1 | 0 | 1 | 0 |
| Amiodarone | 44 | 13 | 42 | 6 | 47 | 13 | 43 | 5 | 43 | 6 | 41 | 11 | 40 | 11 |  | 106 | 24 | 107 | 28 | 98 | 36 | 97 | 21 | 101 | 26 | 99 | 21 | 102 | 25 |
| Amitriptyline | 1 | 0 | 1 | 0 | 1 | 1 | 1 | 0 | 1 | 0 | 1 | 0 | 1 | 1 |  | 78 | 28 | 74 | 22 | 72 | 32 | 69 | 15 | 104 | 26 | 81 | 38 | 70 | 27 |
| Amlodipine | 1 | 0 | 1 | 0 | 1 | 0 | 1 | 1 | 1 | 1 | 1 | 1 | 1 | 0 |  | 94 | 47 | 98 | 44 | 123 | 90 | 110 | 32 | 143 | 59 | 112 | 28 | 114 | 61 |
| Antipyrine | 29 | 3 | 1 | 1 | 1 | 0 | 1 | 1 | 1 | 0 | 1 | 1 | 1 | 0 |  | 34 | 2 | 1 | 1 | 2 | 1 | 2 | 1 | 2 | 1 | 1 | 1 | 2 | 1 |
| Atrazine | 18 | 4 | 0 | 0 | 0 | 0 | 0 | 0 | 0 | 0 | 0 | 0 | 0 | 0 |  | 16 | 1 | 0 | 0 | 1 | 0 | 0 | 0 | 0 | 0 | 0 | 0 | 0 | 0 |
| Azelnidipine | nd | nd | nd | nd | nd | nd | nd | nd | nd | nd | nd | nd | nd | nd |  | nd | nd | nd | nd | nd | nd | nd | nd | nd | nd | nd | nd | nd | nd |
| Azithromycin | nd | nd | nd | nd | nd | nd | nd | nd | nd | nd | nd | nd | nd | nd |  | nd | nd | nd | nd | nd | nd | nd | nd | nd | nd | nd | nd | nd | nd |
| Azoxystrobin | 1 | 0 | 1 | 0 | 1 | 0 | 1 | 0 | 1 | 0 | 1 | 0 | 1 | 0 |  | 144 | 28 | 123 | 42 | 221 | 96 | 155 | 46 | 149 | 50 | 168 | 62 | 150 | 69 |
| Benoxacor | 23 | 19 | 26 | 12 | 18 | 6 | 20 | 17 | 18 | 11 | 22 | 12 | 14 | 7 |  | 94 | 35 | 91 | 65 | 96 | 94 | 145 | 130 | 107 | 55 | 92 | 64 | 118 | 57 |
| Benzatropine | 0 | 0 | 0 | 0 | 0 | 0 | 0 | 0 | 0 | 0 | 0 | 0 | 0 | 0 |  | 189 | 40 | 175 | 40 | 175 | 21 | 239 | 42 | 205 | 31 | 205 | 14 | 217 | 39 |
| Benzoylecgonine | 29 | 4 | 0 | 0 | 1 | 0 | 1 | 0 | 0 | 0 | 0 | 0 | 0 | 0 |  | 20 | 2 | 0 | 0 | 0 | 0 | 0 | 0 | 0 | 0 | 0 | 0 | 0 | 0 |
| Betaxolol | nd | nd | nd | nd | nd | nd | nd | nd | nd | nd | nd | nd | nd | nd |  | nd | nd | nd | nd | nd | nd | nd | nd | nd | nd | nd | nd | nd | nd |
| Bezafibrate | 1 | 1 | 0 | 0 | 1 | 0 | 1 | 0 | 1 | 0 | 0 | 0 | 1 | 0 |  | 1 | 0 | 1 | 1 | 1 | 0 | 1 | 0 | 1 | 0 | 1 | 0 | 0 | 0 |
| Bisoprolol | 2 | 1 | 0 | 0 | 0 | 0 | 0 | 0 | 0 | 0 | 0 | 0 | 0 | 0 |  | 10 | 2 | 0 | 0 | 0 | 0 | 0 | 0 | 0 | 0 | 0 | 0 | 0 | 0 |
| Bupropion | 11 | 4 | 0 | 0 | 11 | 3 | 1 | 0 | 0 | 0 | 0 | 0 | 0 | 0 |  | 60 | 7 | 0 | 0 | 2 | 1 | 0 | 0 | 0 | 0 | 0 | 0 | 0 | 0 |
| Buspirone | nd | 0 | 0 | 0 | 0 | 0 | 0 | 0 | 0 | 0 | 0 | 0 | 0 | 0 |  | 37 | 9 | 2 | 1 | 2 | 1 | 2 | 2 | 3 | 2 | 1 | 0 | 2 | 1 |
| Carazolol | 1 | 1 | 0 | 0 | 0 | 0 | 0 | 0 | 0 | 0 | 0 | nd | 0 | 0 |  | 76 | 34 | 125 | 107 | 79 | 29 | 59 | 14 | 88 | 52 | 25 | nd | 49 | 10 |
| Carboxine | 21 | 4 | 0 | 0 | 0 | 0 | 0 | 0 | 1 | 0 | 0 | 0 | 0 | 0 |  | 33 | 12 | 9 | 1 | 10 | 3 | 12 | 4 | 10 | 4 | 14 | 6 | 12 | 4 |
| CBZ epoxide | 19 | 4 | 0 | 0 | 0 | 0 | 0 | 0 | 0 | 0 | 0 | 0 | 0 | 0 |  | 14 | 2 | 0 | 0 | 0 | 0 | 0 | 0 | 0 | 0 | 0 | 0 | 0 | 0 |
| Chloramphenicol | 35 | 16 | 30 | nd | 5 | nd | 5 | nd | 51 | 24 | 5 | nd | 5 | nd |  | 22 | 19 | 28 | nd | 16 | nd | nd | nd | 26 | 20 | 15 | 13 | nd | nd |
| Cilazapril | 1 | 0 | 1 | 0 | 1 | 0 | 1 | 0 | 1 | 0 | 1 | 0 | 1 | 0 |  | 2 | 1 | 1 | 0 | 1 | 0 | 1 | 0 | 1 | 0 | 1 | 0 | 1 | 0 |
| Citalopram | 8 | 2 | 8 | 3 | 8 | 2 | 8 | 1 | 7 | 2 | 7 | 3 | 8 | 2 |  | 79 | 24 | 73 | 9 | 72 | 32 | 68 | 11 | 71 | 17 | 68 | 12 | 59 | 17 |
| Clodinafop-propargyl | 1 | 1 | 1 | 0 | 2 | 1 | 1 | 1 | 2 | 1 | 1 | 0 | 1 | 1 |  | 117 | 66 | 119 | 72 | 93 | 62 | 162 | 118 | 76 | 19 | 216 | 156 | 166 | 97 |
| Clopidogrel | 0 | 0 | 0 | 0 | 0 | 0 | 0 | 0 | 0 | 0 | 0 | 0 | 0 | 0 |  | 341 | 125 | 151 | 44 | 210 | 58 | 201 | 106 | 229 | 138 | 249 | 77 | 83 | 46 |
| Clothianidin | 26 | 6 | nd | nd | nd | nd | 2 | nd | 1 | 0 | nd | nd | nd | nd |  | 28 | 12 | 2 | 1 | nd | nd | nd | nd | 4 | 2 | 1 | nd | 3 | nd |
| Cocaine | 8 | 3 | 0 | 0 | 14 | 3 | 1 | 0 | 0 | 0 | 0 | 0 | 0 | 0 |  | 130 | 14 | 2 | 1 | 4 | 1 | 3 | 1 | 2 | 1 | 1 | 0 | 1 | 0 |
| Diazepam | 3 | 1 | 2 | 1 | 3 | 2 | 2 | 1 | 2 | 1 | 2 | 1 | 1 | 1 |  | 55 | 24 | 59 | 21 | 67 | 29 | 53 | 21 | 51 | 18 | 67 | 29 | 56 | 28 |
| Dimethametryn | 1 | 0 | 0 | 0 | 0 | 0 | 0 | 0 | 0 | 0 | 0 | 0 | 0 | 0 |  | 56 | 38 | 19 | 9 | 15 | 5 | 27 | 16 | 16 | 12 | 15 | 8 | 14 | 4 |
| Diphenhydramine | 3 | 2 | 0 | 0 | 1 | 1 | 0 | 0 | 0 | 0 | 0 | 0 | 0 | 0 |  | 28 | 6 | 0 | 0 | 0 | 0 | 0 | 0 | 1 | 0 | 1 | 0 | 0 | 0 |
| Famoxadone | 1 | 0 | 1 | 0 | 1 | 0 | 1 | 0 | 1 | 0 | 1 | 0 | 1 | 0 |  | nd | nd | nd | nd | nd | nd | nd | nd | nd | nd | nd | nd | nd | nd |
| Fenoxaprop-ethyl | 1 | 0 | 1 | 0 | 1 | 0 | 1 | 0 | 1 | 0 | 1 | 0 | 1 | 0 |  | 109 | 37 | 172 | 66 | 102 | 48 | 130 | 56 | 110 | 52 | 100 | 33 | 104 | 66 |
| Fenuron | 28 | 4 | nd | nd | nd | nd | 0 | nd | 2 | 1 | 0 | 0 | nd | nd |  | 25 | 3 | nd | nd | 0 | 0 | 0 | 0 | 1 | 1 | nd | nd | 0 | nd |
| Flufenoxuron | 64 | 17 | 60 | 26 | 78 | 29 | 63 | 30 | 91 | 37 | 96 | 89 | 62 | 36 |  | 77 | 32 | 127 | 35 | 90 | 47 | 107 | 64 | 95 | 32 | 96 | 36 | 84 | 27 |
| Fluocinonide | 42 | 11 | 49 | 18 | 45 | 7 | 52 | 18 | 49 | 11 | 39 | 11 | 50 | 19 |  | 108 | 49 | 100 | 24 | 81 | 25 | 97 | 37 | 114 | 44 | 103 | 25 | 93 | 33 |
| Fluoxetine | 12 | 2 | 12 | 2 | 11 | 1 | 11 | 1 | 11 | 2 | 12 | 2 | 11 | 1 |  | 95 | 13 | 86 | 10 | 87 | 18 | 94 | 15 | 85 | 9 | 95 | 15 | 88 | 16 |
| Flutolanil | 1 | 1 | 1 | 0 | 1 | 0 | 1 | 0 | 1 | 0 | 1 | 0 | 1 | 0 |  | 79 | 30 | 59 | 24 | 109 | 50 | 82 | 42 | 93 | 32 | 72 | 35 | 88 | 53 |
| Indomethacin | 55 | 9 | 52 | 12 | 50 | 11 | 53 | 11 | 44 | 8 | 42 | 10 | 52 | 9 |  | 115 | 32 | 101 | 28 | 105 | 28 | 118 | 22 | 89 | 31 | 88 | 21 | 80 | 27 |
| Isocarbamid | 27 | 5 | nd | nd | nd | nd | nd | nd | 4 | 2 | nd | nd | nd | nd |  | 30 | 9 | 1 | nd | 2 | 1 | nd | nd | 3 | 2 | nd | nd | 2 | nd |
| Josamycin | 1 | 1 | 1 | 0 | 1 | 1 | 1 | 0 | 1 | 0 | 1 | 0 | 1 | 1 |  | 108 | 33 | 74 | 46 | 93 | 33 | 94 | 45 | 98 | 29 | 87 | 56 | 108 | 28 |
| Ketamine | 20 | 5 | 0 | 0 | 20 | 6 | 2 | 1 | 0 | 0 | 0 | 0 | 0 | 0 |  | 43 | 5 | 0 | 0 | 3 | 2 | 1 | 0 | 0 | 0 | 0 | 0 | 0 | 0 |
| Ketotifen | 1 | 0 | 0 | 0 | 0 | 0 | 0 | 0 | 0 | 0 | 0 | 0 | 0 | 0 |  | 927 | 90 | 102 | 32 | 94 | 38 | 69 | 25 | 68 | 25 | 73 | 20 | 89 | 23 |
| Meclizine | 2 | 1 | 2 | 1 | 3 | 2 | 2 | 1 | 2 | 0 | 2 | 1 | 2 | 1 |  | 93 | 26 | 81 | 34 | 91 | 42 | 76 | 33 | 87 | 40 | 100 | 57 | 80 | 34 |
| Meclofenamic acid | 88 | 21 | 93 | 29 | 108 | 28 | 114 | 41 | 85 | 19 | 78 | 24 | 92 | 34 |  | 80 | 31 | 87 | 26 | 87 | 32 | 74 | 20 | 79 | 20 | 88 | 29 | 92 | 34 |
| Medroxy-  progesterone | 13 | 3 | 12 | 7 | 13 | 5 | 12 | 3 | 12 | 3 | 12 | 4 | 12 | 2 |  | 92 | 31 | 98 | 35 | 110 | 49 | 100 | 46 | 127 | 58 | 94 | 11 | 105 | 36 |
| Mefenamic acid | 3 | 1 | 2 | 1 | 2 | 1 | 3 | 1 | 2 | 1 | 2 | 1 | 2 | 1 |  | 74 | 20 | 79 | 21 | 60 | 12 | 78 | 18 | 64 | 19 | 78 | 22 | 72 | 26 |
| Memantine | 12 | 4 | 0 | 0 | 18 | 3 | 5 | 1 | 0 | 0 | 0 | 0 | 0 | 0 |  | 25 | 4 | 1 | 0 | 4 | 1 | 1 | 0 | 0 | 0 | 0 | 0 | 0 | 0 |
| Mephedrone | 28 | 3 | 0 | 0 | 37 | 2 | 12 | 1 | 0 | 0 | 0 | 0 | 0 | 0 |  | 96 | 10 | 0 | 0 | 32 | 14 | 9 | 2 | 0 | 0 | 0 | 0 | 0 | 0 |
| Mephosfolan | 25 | 5 | 0 | 0 | 0 | 0 | 0 | 0 | 0 | 0 | 0 | 0 | 0 | 0 |  | 24 | 3 | 0 | 0 | 0 | 0 | 0 | 0 | 0 | 0 | 0 | 0 | 0 | 0 |
| Methedrone | 27 | 3 | 1 | 0 | 40 | 4 | 14 | 2 | 1 | 0 | 0 | 0 | 1 | 0 |  | 36 | 25 | 1 | 0 | 19 | 7 | 6 | 2 | 1 | 1 | 1 | 1 | 1 | 1 |
| Methylphenidate | 14 | 4 | 0 | 0 | 35 | 5 | 9 | 1 | 0 | 0 | 0 | 0 | 0 | 0 |  | 246 | 26 | 0 | 0 | 18 | 6 | 8 | 3 | 0 | 0 | 0 | 0 | 0 | 0 |
| Metoprolol | 16 | 5 | 0 | 0 | 9 | 2 | 2 | 1 | 0 | 0 | 0 | 0 | 0 | 0 |  | 33 | 4 | 0 | 0 | 1 | 0 | 1 | 0 | 0 | 0 | 0 | 0 | 0 | 0 |
| Nadolol | 23 | 5 | nd | nd | 37 | 5 | 15 | 2 | nd | nd | 0 | nd | nd | nd |  | 16 | 3 | nd | nd | 5 | 1 | 2 | 1 | 0 | nd | nd | nd | nd | nd |
| Nicotine | 32 | 3 | nd | nd | 40 | 2 | 3 | 1 | 0 | 0 | 0 | 0 | 1 | 0 |  | nd | nd | nd | nd | 40 | 14 | 15 | 8 | 0 | 0 | 0 | 0 | 2 | 0 |
| Nitenpyram | 27 | 2 | 1 | 0 | 1 | 0 | 1 | 0 | 1 | 0 | 1 | 1 | 1 | 0 |  | 28 | 14 | 1 | 1 | 1 | 0 | 1 | 0 | 2 | 1 | 1 | 0 | 1 | 0 |
| Norethisterone | nd | nd | nd | nd | nd | nd | nd | nd | nd | nd | nd | nd | nd | nd |  | 92 | 16 | 86 | 30 | 82 | 25 | 100 | 29 | 69 | 21 | 78 | 26 | 91 | 24 |
| Nortriptyline | nd | nd | nd | nd | nd | nd | nd | nd | nd | nd | nd | nd | nd | nd |  | 74 | 40 | 102 | 68 | 99 | 36 | 141 | 49 | 103 | 58 | 101 | 38 | 137 | 106 |
| Orphenadrine | 1 | 0 | 0 | 0 | 0 | 0 | 0 | 0 | 0 | 0 | 0 | 0 | 0 | 0 |  | 26 | 4 | 2 | 1 | 2 | 1 | 2 | 1 | 3 | 1 | 3 | 1 | 2 | 1 |
| Oxamyl | 15 | 4 | nd | nd | nd | nd | 0 | 0 | 2 | 1 | nd | nd | nd | nd |  | 48 | 10 | nd | nd | 0 | nd | 1 | 0 | 7 | 2 | nd | nd | nd | nd |
| Oxycarboxin | 24 | 5 | nd | nd | 1 | nd | nd | nd | 7 | 2 | nd | nd | nd | nd |  | 26 | 6 | 0 | nd | 1 | nd | 1 | 0 | 4 | 1 | nd | nd | 1 | nd |
| Oxycodone | 38 | 6 | 0 | 0 | 47 | 4 | 17 | 2 | 0 | 0 | 0 | 0 | 0 | 0 |  | 65 | 6 | 0 | 0 | 11 | 4 | 5 | 1 | 0 | 0 | 0 | 0 | 0 | 0 |
| Picoxystrobin | 4 | 1 | 3 | 1 | 3 | 1 | 3 | 1 | 3 | 1 | 4 | 2 | 4 | 1 |  | nd | nd | nd | nd | nd | nd | nd | nd | nd | nd | nd | nd | nd | nd |
| Piperophos | 1 | 0 | 0 | 0 | 1 | 0 | 0 | 0 | 0 | 0 | 0 | 0 | 0 | 0 |  | nd | nd | nd | nd | nd | nd | nd | nd | nd | nd | nd | nd | nd | nd |
| Pirenzipine | 24 | 5 | 1 | 1 | 31 | 2 | 11 | 2 | 1 | 1 | 1 | 0 | 1 | 1 |  | 21 | 3 | 1 | 0 | 6 | 2 | 3 | 1 | 1 | 0 | 1 | 0 | 1 | 0 |
| Pretilachlor | 2 | 0 | 1 | 1 | 1 | 1 | 2 | 1 | 2 | 0 | 1 | 1 | 2 | 0 |  | 86 | 45 | 88 | 11 | 71 | 26 | 78 | 32 | 80 | 24 | 97 | 30 | 84 | 26 |
| Prometon | 11 | 4 | 0 | 0 | 0 | 0 | 0 | 0 | 0 | 0 | 0 | 0 | 0 | 0 |  | 19 | 4 | 0 | 0 | 0 | 0 | 0 | 0 | 0 | 0 | 0 | 0 | 0 | 0 |
| Prometryn | 2 | 1 | 0 | 0 | 0 | 0 | 0 | 0 | 0 | 0 | 0 | 0 | 0 | 0 |  | 35 | 6 | 1 | 0 | 1 | 1 | 1 | 1 | 1 | 0 | 1 | 1 | 1 | 0 |
| Propanolol | 2 | 1 | 1 | 0 | 0 | 0 | 1 | 0 | 0 | 0 | 0 | 0 | 0 | 0 |  | 24 | 4 | 1 | 1 | 1 | 0 | 2 | 1 | 2 | 1 | 2 | 1 | 2 | 1 |
| Propazine | 10 | 4 | 0 | 0 | 0 | 0 | 0 | 0 | 0 | 0 | 0 | 0 | 0 | 0 |  | 8 | 1 | 1 | 0 | 1 | 0 | 1 | 1 | 1 | 0 | 1 | 0 | 1 | 0 |
| Pymetrozine | 27 | 4 | 0 | 0 | 1 | 1 | 1 | 0 | 0 | 0 | 0 | 0 | 0 | 0 |  | 28 | 8 | 1 | 0 | 1 | 1 | 1 | 0 | 0 | 0 | 0 | 0 | 1 | 0 |
| Pyracarbolid | 24 | 4 | 1 | 0 | 1 | 0 | 1 | 0 | 1 | 0 | 0 | 0 | 1 | 0 |  | 20 | 4 | 2 | 1 | 2 | 0 | 2 | 1 | 2 | 0 | 2 | 1 | 2 | 1 |
| Pyraclostrobin | 4 | 2 | 3 | 1 | 3 | 3 | 3 | 1 | 3 | 2 | 3 | 2 | 3 | 2 |  | 138 | 71 | 188 | 96 | 127 | 69 | 158 | 91 | 142 | 71 | 116 | 36 | 198 | 131 |
| Pyraflufen-ethyl | 5 | 1 | 5 | 2 | 5 | 2 | 6 | 2 | 4 | 1 | 5 | 2 | 6 | 2 |  | 121 | 38 | 121 | 34 | 119 | 32 | 123 | 36 | 84 | 9 | 105 | 36 | 107 | 34 |
| Pyridaben | 65 | 18 | 89 | 22 | 80 | 13 | 94 | 19 | 77 | 10 | 69 | 13 | 82 | 15 |  | 106 | 31 | 98 | 24 | 91 | 18 | 96 | 23 | 83 | 18 | 94 | 20 | 105 | 31 |
| Risperidone | 1 | 0 | 0 | 0 | 0 | 0 | 0 | 0 | 0 | 0 | 0 | 0 | 0 | 0 |  | 31 | 14 | 12 | 5 | 20 | 6 | 15 | 7 | 14 | 5 | 11 | 2 | 13 | 4 |
| Ronidazole | 23 | 2 | 0 | 0 | 1 | 0 | 1 | 0 | 0 | 0 | 1 | 0 | 0 | 0 |  | 29 | 4 | 1 | 0 | 1 | 0 | 1 | 0 | 1 | 0 | 0 | 0 | 1 | 1 |
| Salicylic acid | 16 | 4 | 16 | 3 | 62 | 19 | 15 | 5 | 46 | 6 | 14 | 6 | 17 | 6 |  | 46 | 13 | 116 | 22 | 200 | 33 | 67 | 12 | 183 | 13 | 40 | 5 | 47 | 14 |
| Sertraline | 1 | 0 | 1 | 0 | 1 | 0 | 1 | 0 | 1 | 0 | 1 | 0 | 1 | 0 |  | 115 | 40 | 121 | 64 | 101 | 30 | 102 | 34 | 90 | 30 | 99 | 45 | 112 | 43 |
| Simazine | 22 | 3 | nd | nd | 1 | nd | 0 | 0 | 0 | 0 | 0 | nd | 1 | 0 |  | 19 | 3 | 0 | 0 | 1 | 0 | 1 | 1 | 0 | 0 | nd | nd | 0 | nd |
| Spinosyn D | nd | nd | nd | nd | nd | nd | nd | nd | nd | nd | nd | nd | nd | nd |  | nd | nd | nd | nd | nd | nd | nd | nd | nd | nd | nd | nd | nd | nd |
| Sulfadimethoxine | 8 | 2 | 1 | 0 | 1 | 0 | 1 | 0 | 1 | 0 | 1 | 0 | 1 | 0 |  | 2 | 0 | 1 | 1 | 1 | 1 | 1 | 0 | 1 | 0 | 1 | 0 | 1 | 1 |
| Sulfamerazine | 5 | 4 | 6 | 6 | 6 | 4 | 4 | 2 | 5 | 3 | 6 | 3 | 4 | 2 |  | 102 | 79 | 89 | 40 | 121 | 49 | 98 | 60 | 107 | 50 | 64 | 56 | 78 | 47 |
| Sulfamethazine | 18 | 4 | 0 | 0 | 0 | 0 | 0 | 0 | 0 | 0 | 0 | 0 | 0 | 0 |  | 24 | 3 | 0 | 0 | 0 | 0 | 0 | 0 | 0 | 0 | 0 | 0 | 0 | 0 |
| Sulfamethoxazole | nd | nd | 1 | 0 | 1 | 0 | 1 | 0 | 1 | 1 | 1 | 0 | 1 | 1 |  | nd | nd | 1 | 0 | 1 | 1 | 1 | 1 | 1 | 1 | 1 | 1 | 1 | 1 |
| Sulfamonomethoxine | 5 | 3 | 1 | 0 | 1 | nd | 0 | nd | 0 | 0 | 2 | nd | 1 | 0 |  | 5 | 2 | 0 | 0 | 0 | 0 | 0 | 0 | 1 | 0 | 1 | 1 | 1 | 0 |
| Sulfapyridine | 29 | 3 | nd | nd | 1 | nd | 0 | 0 | 0 | nd | nd | nd | nd | nd |  | 18 | 3 | 0 | 0 | nd | nd | 0 | nd | 0 | 0 | nd | nd | 0 | nd |
| Sulfathiazole | 9 | 3 | 0 | 0 | 0 | 0 | 0 | 0 | 0 | 0 | 0 | 0 | 0 | 0 |  | 17 | 2 | 0 | 0 | 0 | 0 | 0 | 0 | 0 | 0 | 0 | 0 | 0 | 0 |
| Sulfisoxazole | 6 | 2 | 1 | 0 | 1 | 0 | 1 | 1 | 1 | 0 | 1 | 0 | 1 | 0 |  | 2 | 1 | 1 | 1 | 1 | 0 | 1 | 1 | 1 | 1 | 2 | 1 | 1 | 0 |
| Tacrine | 9 | 4 | 0 | 0 | 9 | 3 | 3 | 1 | 0 | nd | 0 | 0 | 0 | 0 |  | 25 | 4 | nd | nd | 1 | 1 | 1 | 0 | 0 | 0 | nd | nd | 0 | 0 |
| Temazepam | 2 | 1 | 1 | 0 | 1 | 0 | 0 | 0 | 1 | 0 | 1 | 0 | 0 | 0 |  | 5 | 1 | 5 | 2 | 5 | 1 | 7 | 3 | 6 | 3 | 6 | 1 | 6 | 2 |
| Terbutryn | 1 | 0 | 0 | 0 | 0 | 0 | 0 | 0 | 0 | 0 | 0 | 0 | 0 | 0 |  | 217 | 67 | 22 | 12 | 32 | 21 | 21 | 11 | 16 | 10 | 24 | 9 | 15 | 10 |
| Terfenadine | 1 | 0 | 0 | 0 | 0 | 0 | 0 | 0 | 0 | 0 | 0 | 0 | 0 | 0 |  | 98 | 43 | 83 | 32 | 58 | 23 | 78 | 44 | 76 | 31 | 61 | 18 | 58 | 29 |
| Thiacloprid | 25 | 4 | 1 | 1 | 1 | 0 | 1 | 0 | 1 | 1 | 1 | 0 | 1 | 0 |  | 40 | 11 | 10 | 5 | 10 | 2 | 9 | 5 | 8 | 3 | 10 | 4 | 9 | 3 |
| Thiamethoxam | 28 | 5 | 2 | 1 | 4 | 2 | 3 | 2 | 6 | 5 | 2 | 1 | 3 | 2 |  | 22 | 3 | 10 | 5 | 3 | 1 | 17 | 12 | 6 | 2 | 4 | 2 | 57 | 8 |
| Thiazopyr | 0 | 0 | 0 | 0 | 0 | 0 | 0 | 0 | 0 | 0 | 0 | 0 | 0 | 0 |  | 56 | 18 | 96 | 56 | 83 | 27 | 74 | 47 | 81 | 55 | 50 | 18 | 77 | 55 |
| Timolol | 14 | 5 | 0 | 0 | 11 | 2 | 3 | 1 | 0 | 0 | 0 | 0 | 0 | 0 |  | 27 | 4 | 0 | 0 | 1 | 0 | 1 | 0 | 0 | 0 | 0 | 0 | 0 | 0 |
| Trimethoprim | 15 | 4 | 0 | 0 | 13 | 4 | 2 | 1 | 0 | 0 | 0 | 0 | 0 | 0 |  | 37 | 5 | 0 | 0 | 2 | 1 | 1 | 0 | 0 | 0 | 0 | 0 | 0 | 0 |
| Valsartan | 6 | 1 | 6 | 2 | 6 | 1 | 5 | 1 | 5 | 2 | 6 | 2 | 5 | 2 |  | 6 | 2 | 6 | 3 | 6 | 2 | 5 | 1 | 6 | 2 | 6 | 3 | 8 | 5 |
| Venlafaxine | 10 | 4 | 0 | 0 | 9 | 2 | 2 | 0 | 0 | 0 | 0 | 0 | 0 | 0 |  | 25 | 4 | 0 | 0 | 1 | 0 | 0 | 0 | 0 | 0 | 0 | 0 | 0 | 0 |
| Verapamil | 1 | 1 | 1 | 0 | 1 | 0 | 1 | 0 | 1 | 1 | 1 | 0 | 1 | 0 |  | 125 | 33 | 99 | 40 | 122 | 28 | 111 | 38 | 119 | 15 | 98 | 27 | 84 | 20 |
| Warfarin | 9 | 3 | 7 | 3 | 6 | 1 | 7 | 1 | 5 | 2 | 5 | 2 | 6 | 2 |  | 129 | 39 | 103 | 29 | 148 | 55 | 113 | 23 | 114 | 32 | 108 | 34 | 99 | 36 |
| Ziprasidone | 1 | 1 | 1 | 0 | 2 | 1 | 1 | 0 | 1 | 1 | 1 | 1 | 1 | 0 |  | 86 | 32 | 62 | 45 | 87 | 54 | 90 | 66 | 70 | 33 | 100 | 46 | 67 | 36 |
|  |  |  |  |  |  |  |  |  |  |  |  |  |  |  |  |  |  |  |  |  |  |  |  |  |  |  |  |  |  |
| **Summary Statistics** | **Artificial Freshwater (n=3)** | | | | | | | | | | | | | |  | **Untreated Wastewater (n=3)** | | | | | | | | | | | | | |
|  | Alu. | | CN-E | | WAX | | AX | | SCX | | CX | | PEP | |  | Alu. | | CN-E | | WAX | | AX | | SCX | | CX | | PEP | |
|  | % | SD | % | SD | % | SD | % | SD | % | SD | % | SD | % | SD |  | % | SD | % | SD | % | SD | % | SD | % | SD | % | SD | % | SD |
| Maximum | 88 | - | 93 | - | 108 | - | 114 | - | 91 | - | 96 | - | 92 | - |  | 927 | - | 188 | - | 221 | - | 239 | - | 229 | - | 249 | - | 217 | - |
| Minimum | 0 | - | 0 | - | 0 | - | 0 | - | 0 | - | 0 | - | 0 | - |  | 1 | - | 0 | - | 0 | - | 0 | - | 0 | - | 0 | - | 0 | - |
| Median % | 10 | - | 1 | - | 1 | - | 1 | - | 1 | - | 0 | - | 1 | - |  | 42 | - | 5 | - | 11 | - | 7 | - | 4 | - | 6 | - | 2 | - |
| **Average %** | **15** | **16** | **6** | **17** | **11** | **20** | **7** | **18** | **6** | **17** | **5** | **16** | **6** | **16** |  | **72** | **105** | **44** | **53** | **45** | **56** | **44** | **56** | **42** | **56** | **44** | **57** | **41** | **53** |

**Table S4.** Performance data for the optimised direct LC-MS/MS method for all 135 pharmaceuticals, metabolites and pesticides in influent wastewater matrix (sampled from London, UK) and comparison with other similar methods.

| Analyte | Range | Linearity N≥5 | Peak Area Precision  (RSD%), n=6 | | Matrix Effect  (CV%), n=6 | | Inaccuracy  (CV%), n=2  n=6 at 1000 ng L^-1^ | | | LLOD | LLOQ | Other Direct LC-MS/MS Method LLOQs |
| --- | --- | --- | --- | --- | --- | --- | --- | --- | --- | --- | --- | --- |
|  | ng L^-1^ | R^2^ | 100  ng L^-1^ | 1000  ng L^-1^ | 100  ng L^-1^ | 1000  ng L^-1^ | 250  ng L^-1^ | 750  ng L^-1^ | 1000  ng L^-1^ | ng L^-1^ | ng L^-1^ | ng L^-1^ |
| 2-(Thiocyano-methylthio) benzothiazole | 100 - 5000 | 0.9998 | 9 | 1 | 3 | -5 | -11 | -12 | -7 | 2 | 5 | - |
| 4-Methyl-ethcathinone | 50 - 5000 | 0.9998 | 7 | 4 | -8 | -6 | -11 | -6 | -2 | 14 | 46 | - |
| Acetamiprid | 50 - 5000 | 0.9991 | 3 | 4 | -2 | -6 | -12 | -10 | -11 | 31 | 102 | 20^a^ |
| Alprazolam | 50 - 5000 | 0.9994 | 23 | 4 | 17 | 8 | 6 | -1 | 5 | 36 | 120 | 10^b^ |
| Ametryn | 25 - 5000 | 0.9997 | 2 | 4 | -9 | -4 | -11 | -7 | -2 | 7 | 24 | - |
| Amiodarone | 250 - 5000 | 0.9980 | n.d. | 5 | -11 | -17 | 2 | -10 | -8 | 141 | 471 | - |
| Amitriptyline | 25 - 5000 | 0.9998 | 12 | 15 | -30 | -10 | 0 | 7 | 1 | 19 | 63 | 10^a^, 10^b^ |
| Amlodipine | 25 - 5000 | 0.9985 | 13 | 8 | 16 | -3 | 7 | -6 | -3 | 1 | 3 | - |
| Antipyrine | 250 - 5000 | 0.9991 | 9 | 5 | -84 | -30 | 66 | 13 | 2 | 54 | 181 | 30^a^ |
| Atorvastatin | 100 - 5000 | 0.9996 | 3 | 6 | 14 | 8 | -5 | 2 | 2 | 70 | 233 | 10^b^ |
| Atrazine | 10 - 5000 | 0.9997 | 5 | 5 | -8 | -2 | -11 | -6 | -3 | 1 | 2 | 10^a^ |
| Azelnidipine | 5 – 5000 | 0.9988 | 6 | 9 | 126 | 80 | 1 | 8 | 7 | 3 | 10 | - |
| Azithromycin | 75 - 5000 | 0.9998 | 11 | 8 | 23 | 146 | 5 | -1 | 7 | 50 | 167 | 15^a^ |
| Azoxystrobin | 50 - 5000 | 0.9990 | 11 | 6 | 10 | 16 | -9 | -10 | -7 | 6 | 19 | 20^a^ |
| Benoxacor | 500 - 5000 | 0.9987 | n.d. | 19 | 8 | 7 | 25 | -6 | 3 | 101 | 339 | - |
| Bensulide | 75 - 5000 | 0.9985 | 20 | 11 | -5 | 7 | 1 | -15 | 1 | 22 | 74 | - |
| Benzatropine | 10 - 5000 | 0.9995 | 7 | 5 | -5 | 0 | -32 | -11 | -3 | 8 | 28 | - |
| Benzoylecgonine* | 500 - 5000 | 0.9950 | 3 | 10 | -36 | 14 | -65 | -14 | -19 | 17 | 57 | - |
| Betaxolol* | 25 - 5000 | 0.9990 | 10 | 13 | -4 | 8 | -12 | 2 | 3 | 14 | 45 | - |
| Bezafibrate | 50 - 5000 | 0.9994 | 6 | 4 | 14 | 4 | -12 | -5 | -15 | 29 | 97 | 10^a^, 10^b^, 5^c^ |
| Bisoprolol | 5 – 5000 | 0.9994 | 4 | 3 | -3 | 3 | -13 | -11 | -7 | 2 | 5 | - |
| Bupropion | 10 - 5000 | 0.9996 | 8 | 4 | -14 | -13 | -12 | -7 | 1 | 7 | 23 | 10^b^ |
| Buspirone | 5 – 5000 | 0.9989 | 6 | 6 | -5 | -2 | -12 | -6 | -4 | 0.4 | 1 | - |
| Carazolol | 5 – 5000 | 0.999 | 6 | 5 | -10 | -10 | -18 | -10 | -11 | 1 | 2 | - |
| Carbamazepine | 10 - 5000 | 0.9968 | 5 | 3 | -14 | -10 | -6 | -16 | -28 | 6 | 20 | 10^a^, 10^b^, 5^c^ |
| Carboxine | 25 - 5000 | 0.9995 | 3 | 4 | -11 | -7 | -11 | -9 | 1 | 2 | 7 | - |
| Carfentrazone-ethyl | 500 - 5000 | 0.9939 | n.d. | 22 | -26 | -13 | 21 | -27 | -3 | 533 | 1777 | - |
| Carbamazepine epoxide | 10 - 5000 | 0.9987 | 9 | 3 | -17 | -10 | -18 | -10 | -19 | 9 | 30 | 10^a^, 10^b^ |
| Celecoxib* | 10 - 5000 | 0.9996 | 27 | 7 | 11 | 20 | -10 | -2 | -19 | 3 | 12 | - |
| Chloramphenicol | 50 - 5000 | 0.9982 | 44 | 10 | 2 | 6 | -22 | -7 | -10 | 34 | 112 | 10^b^ |
| Cilazapril | 10 - 5000 | 0.9996 | 10 | 5 | 5 | 7 | -16 | -6 | 1 | 1 | 5 | - |
| Citalopram | 50 - 5000 | 0.9991 | 3 | 6 | -22 | -18 | -15 | -11 | -1 | 21 | 68 | 10^a^, 15^c^ |
| Clarithromycin* | 75 - 5000 | 0.9922 | 7 | 15 | -57 | -4 | -40 | -11 | -7 | 40 | 135 | 10^a^,10^b^, 10^c^ |
| Clodinafop-propargyl | 25 - 5000 | 0.9983 | 12 | 6 | -38 | -60 | 9 | -10 | -12 | 3 | 9 | - |
| Clopidogrel | 25 - 5000 | 0.9994 | 5 | 4 | -10 | -8 | -14 | -9 | -7 | 10 | 33 | 0.5^c^ |
| Clothianidin* | 250 - 5000 | 0.9748 | n.d. | 19 | -47 | 8 | -57 | -28 | -1 | 61 | 204 | - |
| Clozapine | 25 - 5000 | 0.9991 | 6 | 4 | 5 | 3 | -6 | -7 | 3 | 0 | 1 | - |
| Cocaine* | 25 - 5000 | 0.9975 | 3 | 15 | 45 | 21 | -18 | 0 | -36 | 14 | 47 | - |
| Cyclouron | 250 - 5000 | 0.9997 | n.d. | 4 | -17 | -15 | 11 | 3 | -3 | 72 | 239 | - |
| Cycloxyidim | 75 - 5000 | 0.9992 | 6 | 5 | -4 | 3 | -22 | -10 | n.a. | 17 | 55 | - |
| Cymoxanil | 750 – 5000 (n=4) | 0.9991 | n.d. | 18 | n.d. | -9 | n.d. | -8 | n.a. | 217 | 724 | - |
| Diazepam* | 50 - 5000 | 0.9966 | 20 | 14 | 12 | 3 | -47 | -9 | n.a. | 3 | 12 | 10^a^, 10^b^ |
| Diclofenac | 75 - 5000 | 0.9991 | 10 | 5 | -41 | -8 | 0 | -9 | -7 | 36 | 120 | 10^b^, 5^c^ |
| Diflubenzuron | 50 - 5000 | 0.9992 | 52 | 8 | -29 | 14 | -31 | 13 | 3 | 23 | 77 | - |
| Dimethametryn | 5 – 5000 | 0.9998 | 7 | 6 | -10 | -9 | -8 | -5 | 2 | 2 | 8 | - |
| Diphenhydramine | 50 - 5000 | 0.9993 | 6 | 8 | -26 | -13 | -18 | -9 | -7 | 2 | 6 | 10^c^ |
| Famoxadone | 10 - 5000 | 0.9997 | 7 | 4 | -49 | -45 | -9 | -6 | -1 | 5 | 17 | - |
| Fenoxaprop-ethyl | 25 - 5000 | 0.9994 | 7 | 5 | -32 | -37 | -5 | -8 | -4 | 11 | 37 | - |
| Fenuron | 25 - 5000 | 0.9980 | 7 | 5 | -24 | -23 | -13 | -11 | -11 | 15 | 50 | - |
| Flufenoxuron | 100 - 5000 | 0.9988 | 31 | 9 | -2 | -7 | -17 | -8 | -6 | 10 | 32 | - |
| Fluocinonide | 250 - 5000 | 0.9986 | n.d. | 15 | 1 | -6 | -15 | 6 | -6 | 121 | 404 | 10^b^ |
| Fluoxetine* | 5 - 5000 | 0.9986 | 9 | 14 | -14 | -6 | -8 | 0 | 5 | 3 | 11 | 20^a^, 10^b^ |
| Flutamide | 5 - 5000 | 0.9992 | 6 | 4 | -16 | -8 | -10 | -10 | -4 | 3 | 9 | - |
| Flutolanil | 50 - 5000 | 0.9996 | 18 | 8 | 9 | -5 | -9 | -3 | 1 | 10 | 34 | - |
| Fuberidazole | 25 - 5000 | 0.9996 | 6 | 6 | -17 | -13 | -13 | -7 | -6 | 6 | 19 | - |
| Hydrochloro-thiazide | 50 - 5000 | 0.9990 | 9 | 9 | -8 | -22 | -13 | -7 | 1 | 19 | 62 | 10^a^, 10^b^, 15^c^ |
| Imidacloprid | 500 - 5000 | 0.9984 | n.d. | 17 | 89 | 15 | -33 | -9 | -22 | 75 | 248 | 10^a^, 15^c^ |
| Indomethacin | 1000 – 5000 (n=3) | 0.9966 | n.d. | 15 | n.d. | -14 | n.d. | 9 | -26 | 131 | 436 | 10^a^, 25^b^ |
| Isocarbamid | 100 - 5000 | 0.9898 | 55 | 23 | -28 | -10 | -43 | -10 | -6 | 132 | 440 | - |
| Isradipine | 75 - 5000 | 0.9991 | 42 | 8 | -38 | 3 | -22 | -4 | 2 | 30 | 99 | - |
| Josamycin | 10 - 5000 | 0.9977 | 9 | 11 | -2 | -7 | -13 | -19 | -13 | 3 | 10 | - |
| Ketamine* | 25 - 5000 | 0.9984 | 4 | 14 | 9 | 10 | -31 | -4 | -4 | 6 | 22 | - |
| Ketoconazole | 100 - 5000 | 0.9980 | 21 | 5 | -17 | 10 | -25 | -7 | 1 | 60 | 199 | - |
| Ketotifen | 25 - 5000 | 0.9988 | 4 | 4 | -6 | -6 | -10 | -11 | -14 | 4 | 12 | - |
| Levamisole | 500 - 5000 | 0.9992 | n.d. | 12 | n.d. | 10 | n.d. | -5 | -7 | 13 | 44 | - |
| Levocabastine | 100 - 5000 | 0.9996 | 8 | 3 | 2 | 2 | -16 | 1 | -1 | 21 | 70 | - |
| Lidocaine* | 5 - 5000 | 0.9983 | 4 | 13 | 4 | 8 | -17 | 0 | -3 | 9 | 30 | 10^b^, 10^c^ |
| MDMA* | 50 - 5000 | 0.9988 | 6 | 11 | 1 | 11 | -21 | -6 | -9 | 13 | 44 | - |
| Meclizine | 5 - 5000 | 0.9991 | 8 | 7 | 15 | -5 | -3 | -10 | -5 | 3 | 12 | - |
| Medroxy-progesterone | 75 - 5000 | 0.9989 | 12 | 4 | 7 | 7 | -4 | -12 | -6 | 31 | 103 | - |
| Mefenamic acid | 25 - 5000 | 0.9992 | 8 | 7 | -31 | -5 | -8 | -6 | -1 | 7 | 24 | 10^a^, 10^b^ |
| Memantine | 25 - 5000 | 0.9995 | 6 | 5 | 9 | -3 | -10 | -11 | -6 | 0 | 0.2 | - |
| Mephedrone | 5 - 5000 | 0.9996 | 3 | 3 | -10 | -4 | -7 | -8 | -4 | 1 | 3 | - |
| Mephosfolan | 10 - 5000 | 0.9980 | 2 | 3 | -18 | -8 | -17 | -11 | -14 | 3 | 9 | - |
| Methamphet-amine | 50 - 5000 | 0.9992 | 3 | 4 | -23 | 6 | -3 | -2 | -2 | 4 | 12 | - |
| Methedrone | 100 - 5000 | 0.9984 | 8 | 4 | -17 | -12 | -2 | -10 | -23 | 38 | 126 | - |
| Methylphenidate* | 25 - 5000 | 0.9985 | 7 | 17 | 10 | 14 | -27 | -5 | -3 | 3 | 11 | 10^b^ |
| Metoprolol* | 25 - 5000 | 0.9971 | 8 | 14 | -11 | 2 | -17 | -5 | 2 | 2 | 6 | 20^a^, 10^b^, 20^c^ |
| Nadolol | 25 - 5000 | 0.9996 | 6 | 5 | -11 | 5 | -13 | -9 | -10 | 10 | 33 | 10^a^, 10^b^ |
| Nifedipine* | 500 - 5000 | 0.9673 | n.d. | 27 | -29 | 24 | -97 | -23 | -6 | 216 | 721 | 10^b^ |
| Nordiazepam* | 100 - 5000 | 0.9905 | 22 | 22 | -2 | -9 | -50 | -12 | -1 | 56 | 185 | - |
| Norethisterone | 750 – 5000 (n=4) | 0.9999 | n.d. | 6 | n.d. | 17 | n.d. | -54 | -35 | 29 | 97 | - |
| Nortriptyline* | 25 - 5000 | 0.9969 | 5 | 15 | -18 | -10 | -9 | 4 | 7 | 13 | 44 | - |
| Orphenadrine | 5 - 5000 | 0.9992 | 4 | 4 | -6 | -2 | -17 | -13 | -2 | 3 | 9 | - |
| Oxamyl | 25 - 5000 | 0.9975 | 16 | 5 | 96 | 62 | -5 | -18 | -29 | 14 | 47 | - |
| Oxazepam* | 25 - 5000 | 0.9998 | 16 | 18 | 29 | 19 | -19 | 4 | -10 | 22 | 73 | 25^b^, 5^c^ |
| Oxycarboxin | 10 - 5000 | 0.9985 | 16 | 6 | 18 | 10 | -12 | -15 | -16 | 0.06 | 0.21 | - |
| Oxycodone | 50 - 5000 | 0.9984 | 5 | 3 | -32 | -31 | -16 | -6 | -10 | 4 | 12 | 10^b^ |
| Picoxystrobin | 50 - 5000 | 0.9991 | 12 | 6 | -9 | -7 | -12 | -6 | 1 | 19 | 63 | - |
| Piperophos | 10 - 5000 | 0.9992 | 11 | 4 | -24 | -19 | -8 | -9 | 0 | 9 | 29 | - |
| Pirenzipine | 5 - 5000 | 0.9982 | 7 | 5 | -8 | -9 | -17 | -19 | -15 | 4 | 13 | - |
| Pretilachlor | 50 - 5000 | 0.9994 | 11 | 5 | 3 | 15 | -10 | -8 | -1 | 18 | 61 | - |
| Prodiamine | 750 – 5000 (n=4) | 0.9939 | n.d. | 32 | n.d. | -15 | n.d. | -22 | 7 | 184 | 613 | - |
| Prometon | 10 - 5000 | 0.9996 | 4 | 4 | -7 | -7 | -12 | -6 | 1 | 1 | 3 | - |
| Prometryn | 25 - 5000 | 0.9998 | 6 | 3 | 4 | -3 | -16 | -5 | -1 | 2 | 6 | - |
| Propranolol | 50 - 5000 | 0.9991 | 4 | 6 | -20 | -3 | 20 | -11 | -11 | 18 | 59 | 20^a^, 10^b^ |
| Propazine | 10 - 5000 | 0.9997 | 8 | 4 | -5 | -5 | -14 | -5 | 1 | 2 | 8 | - |
| Pymetrozine | 100 - 5000 | 0.9991 | 9 | 3 | 6 | -1 | -6 | -7 | -15 | 8 | 27 | - |
| Pyracarbolid | 25 - 5000 | 0.9985 | 7 | 5 | -27 | -15 | -10 | -9 | -11 | 9 | 31 | - |
| Pyraclostrobin | 50 - 5000 | 0.9991 | 20 | 6 | -51 | -36 | -8 | -3 | -2 | 33 | 110 | - |
| Pyraflufen-ethyl | 100 - 5000 | 0.9981 | 20 | 7 | -18 | -29 | -22 | -7 | 6 | 25 | 83 | - |
| Pyridaben | 25 - 5000 | 0.9994 | 8 | 6 | -7 | 0 | -4 | -14 | -16 | 6 | 19 | - |
| Risperidone* | 25 - 5000 | 0.9989 | 5 | 13 | -3 | -6 | -25 | -14 | -10 | 2 | 7 | - |
| Rizatripam | 50 - 5000 | 0.9915 | 6 | 6 | -5 | -14 | -20 | -35 | -44 | 1 | 4 | - |
| Ronidazole | 100 - 5000 | 0.9995 | 12 | 7 | -28 | -24 | -41 | -12 | -1 | 26 | 87 | - |
| Roxithromycin | 5 - 5000 | 0.9993 | 8 | 5 | 10 | 7 | -19 | -5 | 0 | 3 | 12 | - |
| Sertraline* | 25 - 5000 | 0.9985 | 15 | 12 | 3 | -9 | -11 | -4 | 5 | 13 | 42 | 10^b^ |
| Simazine | 25 - 5000 | 0.9995 | 12 | 8 | -13 | -6 | -3 | -4 | 0 | 10 | 32 | 20^a^ |
| Spinosyn A | 25 - 5000 | 0.9991 | 14 | 7 | 2 | -10 | -14 | -6 | 0 | 9 | 29 | - |
| Spinosyn D | 50 - 5000 | 0.9984 | 37 | 8 | 7 | -8 | 15 | -21 | -2 | 6 | 18 | - |
| Spiramycin | 25 - 5000 | 0.9990 | 7 | 7 | 337 | 188 | -35 | -5 | -2 | 1 | 4 | - |
| Sulfadimethoxine | 50 - 5000 | 0.9994 | 17 | 4 | -1 | 7 | -12 | -8 | -8 | 34 | 113 | - |
| Sulfamerazine | 500 - 5000 | 0.9987 | n.d. | 14 | n.d. | -11 | 18 | 4 | -17 | 87 | 291 | 10^b^ |
| Sulfamethazine* | 25 - 5000 | 0.9954 | 8 | 13 | -9 | -5 | -56 | -34 | -37 | 2 | 6 | 20^a^ |
| Sulfamethoxazole | 250 - 5000 | 0.9995 | 32 | 18 | 11 | 24 | -5 | -8 | -18 | 68 | 228 | 20^a^, 10^b^, 20^c^ |
| Sulfamono-methoxine | 50 - 5000 | 0.9998 | 21 | 5 | 18 | 19 | -17 | -1 | -4 | 42 | 138 | - |
| Sulfapyridine | 100 - 5000 | 0.9982 | 5 | 5 | -40 | -15 | -24 | -1 | -26 | 49 | 162 | 20^a^ |
| Sulfathiazole | 100 - 5000 | 0.9975 | 5 | 6 | -38 | -23 | -11 | -1 | -12 | 3 | 11 | 50^a^, 10^b^ |
| Sulfisoxazole | 25 - 5000 | 0.9995 | 8 | 6 | 8 | 7 | -12 | -11 | -1 | 13 | 44 | - |
| Tacrine | 25 - 5000 | 0.9994 | 6 | 4 | -6 | -4 | -12 | -6 | -2 | 5 | 17 | - |
| Tamsulosin | 10 - 5000 | 0.9992 | 5 | 5 | 11 | 15 | -15 | -7 | -7 | 2 | 7 | - |
| Temazepam* | 25 - 5000 | 0.9974 | 7 | 15 | -5 | 2 | -27 | -2 | -1 | 9 | 30 | 10^b^ |
| Terbutryn | 10 - 5000 | 0.9996 | 5 | 3 | -10 | -7 | -20 | -2 | 4 | 5 | 17 | 20^a^, 1^c^ |
| Terfenadine | 10 - 5000 | 0.9997 | 11 | 5 | -12 | -20 | -11 | -10 | -3 | 3 | 9 | - |
| Thiacloprid | 75 - 5000 | 0.9993 | 10 | 6 | 12 | -2 | 2 | -10 | -20 | 1 | 3 | - |
| Thiamethoxam* | 75 - 5000 | 0.9996 | 32 | 16 | -3 | 15 | -3 | -6 | -10 | 25 | 84 | - |
| Thiazopyr | 10 - 5000 | 0.9997 | 12 | 3 | 1 | 0 | -11 | -5 | 5 | 3 | 11 | - |
| Timolol | 5 - 5000 | 0.9983 | 5 | 5 | -20 | -15 | -14 | -11 | -15 | 0.3 | 1 | 10^b^ |
| Tramadol* | 25 - 5000 | 0.9993 | 4 | 12 | -13 | 1 | -12 | -10 | -18 | 6 | 20 | 10^b^, 15^c^ |
| Trimethoprim* | 25 - 5000 | 0.9963 | 8 | 12 | -13 | -5 | 8 | 7 | 1 | 9 | 29 | 20^a^, 10^b^, 10^c^ |
| Valsartan | 500 - 5000 | 0.9994 | 10 | 12 | 57 | 21 | -2 | -4 | -14 | 101 | 337 | 10^b^, 10^c^ |
| Venlafaxine* | 25 - 5000 | 0.9980 | 3 | 13 | 0 | 4 | -10 | 8 | 9 | 4 | 13 | 20^a^, 10^b^, 5^c^ |
| Verapamil* | 5 - 5000 | 0.9960 | 10 | 19 | -5 | -4 | -12 | 0 | 1 | 0 | 1 | - |
| Warfarin | 75 - 5000 | 0.9994 | 14 | 7 | -13 | 4 | -16 | -9 | -6 | 62 | 205 | 10^b^ |
| Ziprasidone | 25 - 5000 | 0.999 | 5 | 6 | -29 | -16 | 4 | -17 | -5 | 14 | 45 | - |

*Corresponding SIL-IS analogue used for peak area ratio-based linearity assessment. All other compounds assessed directly using calibration in background-subtracted matrix-matched standards in pooled London influent wastewater

^a^Campos-Manas et al. LLOQ in effluent wastewater [1], ^b^ Oliveira et al. LLOQ in influent wastewater (lowest LLOQ of both methods presented here) [2], ^c^ Hermes et al. LLOQ data in influent wastewater [3]

n.d. not detected; n.a. not applicable/not included

[1] M.C. Campos-Mañas, P. Plaza-Bolaños, J.A. Sánchez-Pérez, S. Malato, A. Agüera, Fast determination of pesticides and other contaminants of emerging concern in treated wastewater using direct injection coupled to highly sensitive ultra-high performance liquid chromatography-tandem mass spectrometry, Journal of Chromatography A, 1507 (2017) 84-94.

[2] T.S. Oliveira, M. Murphy, N. Mendola, V. Wong, D. Carlson, L. Waring, Characterization of Pharmaceuticals and Personal Care products in hospital effluent and waste water influent/effluent by direct-injection LC-MS-MS, Science of The Total Environment, 518-519 (2015) 459-478.

[3] N. Hermes, K.S. Jewell, A. Wick, T.A. Ternes, Quantification of more than 150 micropollutants including transformation products in aqueous samples by liquid chromatography-tandem mass spectrometry using scheduled multiple reaction monitoring, Journal of Chromatography A, 1531 (2018) 64-73.

**Table S5.** % Instability of all analytes in spiked wastewater (500 ng L^-1^) thawing from frozen over a 48-h period (n=3). nd – not determined. Positive integers represent losses of analyte relative to a freshly thawed spiked wastewater matrix and vice versa for negative values.

| **Analyte** | **Instability (%)** | **SD** | **Analyte** | **Instability (%)** | **SD** | **Analyte** | **Instability (%)** | **SD** |
| --- | --- | --- | --- | --- | --- | --- | --- | --- |
| 2-(Thiocyanomethylthio) benzothiazole | 5 | 9 | Fenoxaprop-ethyl | 58 | 3 | Pretilachlor | -3 | 4 |
| 4-Methylethcathinone | 6 | 7 | Fenuron | -1 | 5 | Prodiamine | 19 | 16 |
| Acetamiprid | 1 | 4 | Flufenoxuron | -9 | 10 | Prometon | 1 | 2 |
| Alprazolam | 0 | 7 | Fluocinonide | 5 | 16 | Prometryn | 1 | 5 |
| Ametryn | -1 | 3 | Fluoxetine | 9 | 5 | Propanolol | -3 | 7 |
| Amiodarone | -17 | 7 | Fluoxetine-d6 | 2 | 14 | Propazine | 3 | 6 |
| Amitriptyline | 1 | 6 | Flutamide | 2 | 9 | Pymetrozine | 0 | 3 |
| Amitriptyline-d3 | 7 | 8 | Flutolanil | 4 | 3 | Pyracarbolid | 3 | 5 |
| Amlodipine | 11 | 8 | Fuberidazole | -1 | 4 | Pyraclostrobin | -6 | 13 |
| Antipyrine | 37 | 4 | Hydrochlorothiazide | 10 | 6 | Pyraflufen-ethyl | -1 | 14 |
| Atorvastatin | -4 | 4 | Imidacloprid | -11 | 13 | Pyridaben | -21 | 27 |
| Atrazine | 1 | 6 | Indomethacin | -3 | 40 | Risperidone | 4 | 3 |
| Azelnidipine | 85 | 2 | Isocarbamid | 1 | 12 | Risperidone-d4 | 6 | 5 |
| Azithromycin | 9 | 6 | Isradipine | -1 | 21 | Rizatriptan | 2 | 3 |
| Azoxystrobin | 1 | 5 | Josamycin | 0 | 5 | Ronidazole | 3 | 9 |
| Benoxacor | -5 | 30 | Ketamine | -3 | 4 | Roxithromycin | 6 | 7 |
| Bensulide | -4 | 9 | Ketamine-d4 | 3 | 4 | Sertraline | 0 | 6 |
| Benzatropine | 0 | 4 | Ketoconazole | 73 | nd | Sertraline-d3 | -2 | 7 |
| Benzoylecgonine | 1 | 3 | Ketotifen | 4 | 5 | Simazine | -8 | 11 |
| Benzoylecgonine-d3 | 7 | 7 | Levamisole | 5 | 18 | Spinosyn A | 7 | 7 |
| Betaxolol | -1 | 3 | Levocabastine | -5 | 16 | Spinosyn D | 5 | 10 |
| Betaxolol-d7 | 6 | 11 | Lidocaine | 1 | 3 | Spiramycin | 7 | 14 |
| Bezafibrate | -3 | 9 | Lidocaine-d10 | 3 | 7 | Sulfadimethoxine | -1 | 5 |
| Bisoprolol | -1 | 7 | MDMA | -1 | 5 | Sulfamerazine | -30 | 51 |
| Bupropion | 5 | 4 | MDMA-d5 | 2 | 6 | Sulfamethazine | 5 | 8 |
| Buspirone | 1 | 4 | Meclizine | -2 | 8 | Sulfamethazine-d4 | 6 | 8 |
| Carazolol | 3 | 7 | Medroxyprogesterone | 0 | 5 | Sulfamethoxazole | 0 | 10 |
| Carbamazepine | 7 | 4 | Mefenamic acid | 8 | 7 | Sulfamonomethoxine | -4 | 8 |
| Carboxine | 7 | 10 | Memantine | -1 | 7 | Sulfapyridine | -3 | 8 |
| Carfentrazone-ethyl | 32 | 19 | Mephedrone | 2 | 6 | Sulfathiazole | 3 | 8 |
| CBZ epoxide | -1 | 4 | Mephosfolan | 1 | 5 | Sulfisoxazole | 9 | 6 |
| Celecoxib | 5 | 16 | Methamphetamine | -2 | 8 | Tacrine | 0 | 6 |
| Celecoxib-d7 | 8 | 10 | Methedrone | 1 | 4 | Tamsulosin | 2 | 4 |
| Chloramphenicol | -28 | 75 | Methylphenidate | 3 | 4 | Temazepam | 0 | 7 |
| Cilazapril | 5 | 5 | Methylphenidate-d9 | 7 | 4 | Temazepam-d5 | 7 | 7 |
| Citalopram | 1 | 6 | Metoprolol | 3 | 5 | Terbutryn | 1 | 5 |
| Clarithromycin | -1 | 5 | Metoprolol-d7 | 6 | 6 | Terfenadine | -1 | 4 |
| Clarithromycin-d3 | 4 | 9 | Nadolol | 1 | 3 | Thiacloprid | 0 | 8 |
| Clodinafop-propargyl | nd | nd | Nifedipine | -45 | 126 | Thiamethoxam | 2 | 3 |
| Clopidogrel | nd | nd | Nifedipine-d4 | 11 | 40 | Thiamethoxam-d3 | -17 | 36 |
| Clothianidin* | nd | nd | Nordiazepam | -2 | 12 | Thiazopyr | 1 | 7 |
| Clozapine | -1 | 6 | Nordiazepam-d5 | nd | nd | Timolol | 0 | 2 |
| Cocaine | 2 | 7 | Norethisterone | 1 | 4 | Tramadol | 1 | 3 |
| Cocaine-d3 | 3 | 9 | Nortriptyline | 0 | 10 | Tramadol-13C1, d3 | 5 | 7 |
| Cyclouron | 2 | 8 | Nortriptyline-d3 | -2 | 8 | Trimethoprim | 0 | 5 |
| Cycloxyidim | 14 | 12 | Orphenadrine | 2 | 9 | Trimethoprim-d3 | 2 | 8 |
| Cymoxanil | -55 | 104 | Oxamyl | 13 | 8 | Valsartan | -34 | 8 |
| Diazepam | -1 | 4 | Oxazepam | 3 | 6 | Venlafaxine | 3 | 5 |
| Diazepam-d6 | 4 | 6 | Oxazepam-d5 | 8 | 8 | Venlafaxine-d6 | 5 | 8 |
| Diclofenac | 3 | 8 | Oxycarboxin | 5 | 6 | Verapamil | 6 | 5 |
| Diflubenzuron | 1 | 6 | Oxycodone | 0 | 4 | Verapamil-d3 | 6 | 8 |
| Dimethametryn | -7 | 7 | Picoxystrobin | -2 | 4 | Warfarin | -20 | 6 |
| Diphenhydramine | 1 | 4 | Piperophos | -4 | 4 | Ziprasidone | 9 | 18 |
| Famoxadone | -4 | 10 | Pirenzipine | -2 | 3 | **Absolute Mean**  **±SD (All)** | **7±12 %** | |

**Figure S1.** Optimisation of flow rate and detection sensitivity for direct LC-MS/MS analysis of London wastewater samples. Both axes shown as logarithmic scales. Injection volume =10 µL; SIL-IS spiking concentration =500 ng L^-1^. Gradient events and run time were systematically altered relative to the change in flow rate used.

**Figure S2.** Van Deemter curve for the 5 x 3 mm Raptor biphenyl (2.6 µm particle size column) using azelnidipine as a probe at 500 ng L^-1^­. Mobile phase was 50:25:25 H_2_O:MeOH:MeCN in 0.1 % formic acid. The optimised flow rate for the LC-MS/MS method was 0.5 mL min^-1^.

**Figure S3.** Direct injection volume optimisation. Panel (a) shows the summed measured signal intensity for all 135 compounds from 0.5-20 µL injection volumes using a 500 ng L^-1^ spiked pooled-wastewater matrix from a London WWTP and (b) shows a box plot of %RSD of measured signal intensities (Note: the line and X represent the median and mean, respectively; boxes represent inter-quartile range; and dots are outliers. An outlier for %RSD at 0.5 µL injection volume at 142 % has been removed for clarity (nordiazepam)). Injections at each volume performed in triplicate.

**Figure S4.** The ESI source before (a) and after (b) running a batch of n=60 wastewater samples

**Figure S5.** Pearson (*R*^2^) and Spearman (*r*) correlations between benzoylecgonine concentrations in wastewater at each location with either cocaine or lidocaine concentrations (each data point represents the measurement of both compounds on the same day). Error bars represent ± one standard deviation of triplicate measurements (see Table 2).
